# Interplay of Superconductivity, Ferromagnetism, and Half-Metallicity in Gated Single-Layer g‑C_3_N_4_

**DOI:** 10.1021/acs.jpclett.5c01013

**Published:** 2025-06-03

**Authors:** Pietro Nicolò Brangi, Francesca Martini, Pierluigi Cudazzo, Matteo Calandra

**Affiliations:** Department of Physics, 19034 University of Trento, Via Sommarive 14, 38123 Povo, Italy

## Abstract

Graphitic carbon nitride (g-C_3_N_4_) hosts lone pairs arising from broken carbon–nitrogen bonds in its heptazine structure. These strongly localized and weakly hybridized states form ultraflat bands, potentially leading to correlated states when doped. Using first-principles calculations, we show that field-effect hole doping in single-layer g-C_3_N_4_ depletes these lone pairs, unveiling a rich phase diagram with a complex interplay of superconducting, half-metallic, and insulating ferromagnetic phases, even at very low charging and in the absence of transition metal ions. Our work highlights gated two-dimensional systems hosting lone pairs as a novel platform for strongly correlated states.

## Graphical abstract

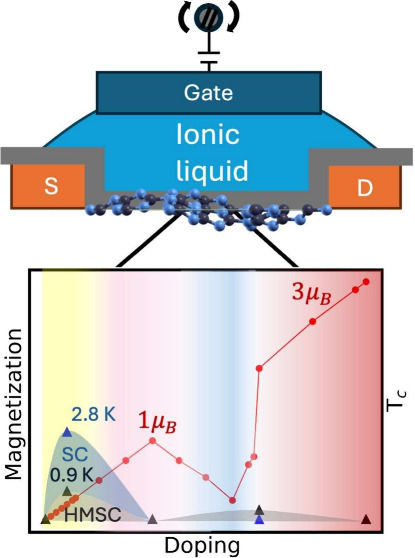

The emergence of strong correlations via the suppression of the single-particle kinetic energy in two-dimensional (2D) materials is a very promising field of research as it can lead to magnetic,^1^ half-metallic,^2^ or superconducting^3,4^ states in the absence of transition metal elements. The two most prominent platforms that have been developed either exploit Moiré-driven flat bands, as in the case of twisted bilayer graphene (TBG),^3^ or rely on ultraflat surface bands, as in the case of rhombohedrally stacked multilayer graphene.^2,5–7^ Despite being successful, these systems result in flat bands that occupy only small portions of the reciprocal space and, as such, host a small number of electrons per atom and do not result in either large magnetic moments or high magnetic or superconducting critical temperatures (*T*_c_). This is the case for multilayer rhombohedral and Bernal graphene, where the largest magnetic moment is of the order of 10^–2^ μ_B_/3 f.u.^2,5–7^ The superconducting *T*_c_ in TBG is of the order of 1 K and, in rhombohedral multilayer graphene, ranges from 100 mK^8–12^ to 1 K.^13^

A different approach that we propose in the current paper is to dope via field-effect (FET) states that are intrinsically strongly localized in real space and naturally result in flat bands in reciprocal space hosting up to ∼3 × 10^15^ electrons/cm^2^. Many localized states can exist in 2D materials; however, the difficulties are to find (i) localized states that weakly hybridize with other more delocalized orbitals and that (ii) lead to flat electronic bands with energy close to the Fermi level. Here, by using density functional theory (DFT),^14,15^ we show that gated 2D graphitic carbon nitride (g-C_3_N_4_) fulfills all of the requirements and that the depleted lone pairs host an interplay between superconducting, half-metallic, and ferromagnetic insulating ground states, in the absence of any transition metal atoms and at very low charging.

Single- and few-layer g-C_3_N_4_ have been under the spotlight for their properties of interest for a large variety of potential applications. The most relevant one is the use as a catalyst for water splitting and hydrogen production but also optoelectronics.^16^ The most interesting characteristic of g-C_3_N_4_ for our purposes lies in the electronic structure: it exhibits an extremely flat top valence band of nitrogen character extending over the whole Brillouin zone.^17^ This band is due to the lone pairs of twofold coordinated nitrogen atoms not involved in σ and π bonding with carbon atoms, and thus its hybridization with other states is weak.

Modifications to the peculiar electronic structure of g-C_3_N_4_ have been studied mainly for what concerns carrier separation to improve the efficiency of the hydrogen evolution process. The possible occurrence of superconductivity has never been investigated. Du et al.^18^ proposed that g-C_4_N_3_ (graphitic carbon nitride where a nitrogen is replaced by a carbon) could display a ferromagnetic half-metallic ground state: there the chemical substitution yields one extra hole for the structure with respect to g-C_3_N_4_. Experimentally, g-C_4_N_3_ was synthesized in 2010,^19^ and only recently it has been shown that g-C_4_N_3_ can be used as a spin thermoelectric material.^20^ However, extensive applications have been hindered by the difficulties related to a complicated synthesis and the lack of tunability of the half-metallic state.^21^ In fact, to our knowledge, no experimental works managed to build g-C_4_N_3_ working devices, although some theoretical works presented possible applications of this material.^20,22,23^ Other works hinted at the possibility of carbon self-doping^24,25^ or transition metal oxide incorporation/coating,^26,27^ leading to a half-metallic state. Such a state was also recently stabilized in nitrogen-doped triangulenes.^28^ However, all of these doping or coating solutions lack the tunability and versatility of field-effect doping. This technique has indeed several advantages: (i) the number of carriers can be varied continuously; (ii) the low carrier concentration region is more accessible; (iii) no chemical constraint, such as wetting or stability conditions, has to be satisfied; (iv) finally, it is an intrinsic doping, free of transition metal or heavy ions. As is clear from the above statement, FET doping is an ideal technique for the exploration of the phase diagram of 2D materials.

In this work, we show that FET doping of single-layer g-C_3_N_4_ leads to a very rich phase diagram displaying a complex interplay of superconducting, half-metallic, and insulating ferromagnetic phases.

We consider the most stable heptazine phase of single-layer g-C_3_N_4_ and perform a complete structural optimization, starting from the ideal flat monolayer, by using the QUANTUM ESPRESSO code^29,30^ (technical details are reported in section S1.1 of the Supporting Information). The flat structure is not the ground state phase of the 2D system as the Coulomb repulsion between the nitrogen lone pairs tends to maximize their distance by corrugating the monolayer.^31–35^ Moreover, it has been pointed out that the 1 × 1 cell might not be the lowest energy structure, even if corrugated.

By optimizing both the flat structure, constraining the atoms in the plane, and the corrugated one, considering different supercells (1 × 1, 2 × 2, and √3 × √3*R*30°), we find that the most stable structure adopts a √3 × √3*R*30° supercell and is highly corrugated, as shown in Figure 1 [see section S3 of the Supporting Information for the calculation, using density functional perturbation theory^36,37^ (DFPT), of the phonon dispersion, which supports our conclusions as already shown in ref 38]. The corrugation can be quantified by evaluating the maximal modulus of the difference between the *z* coordinates of the atoms: we find a corrugation of 2.388 Å (more about the structure is reported in section S1.2 of the Supporting Information).

**Figure 1:**
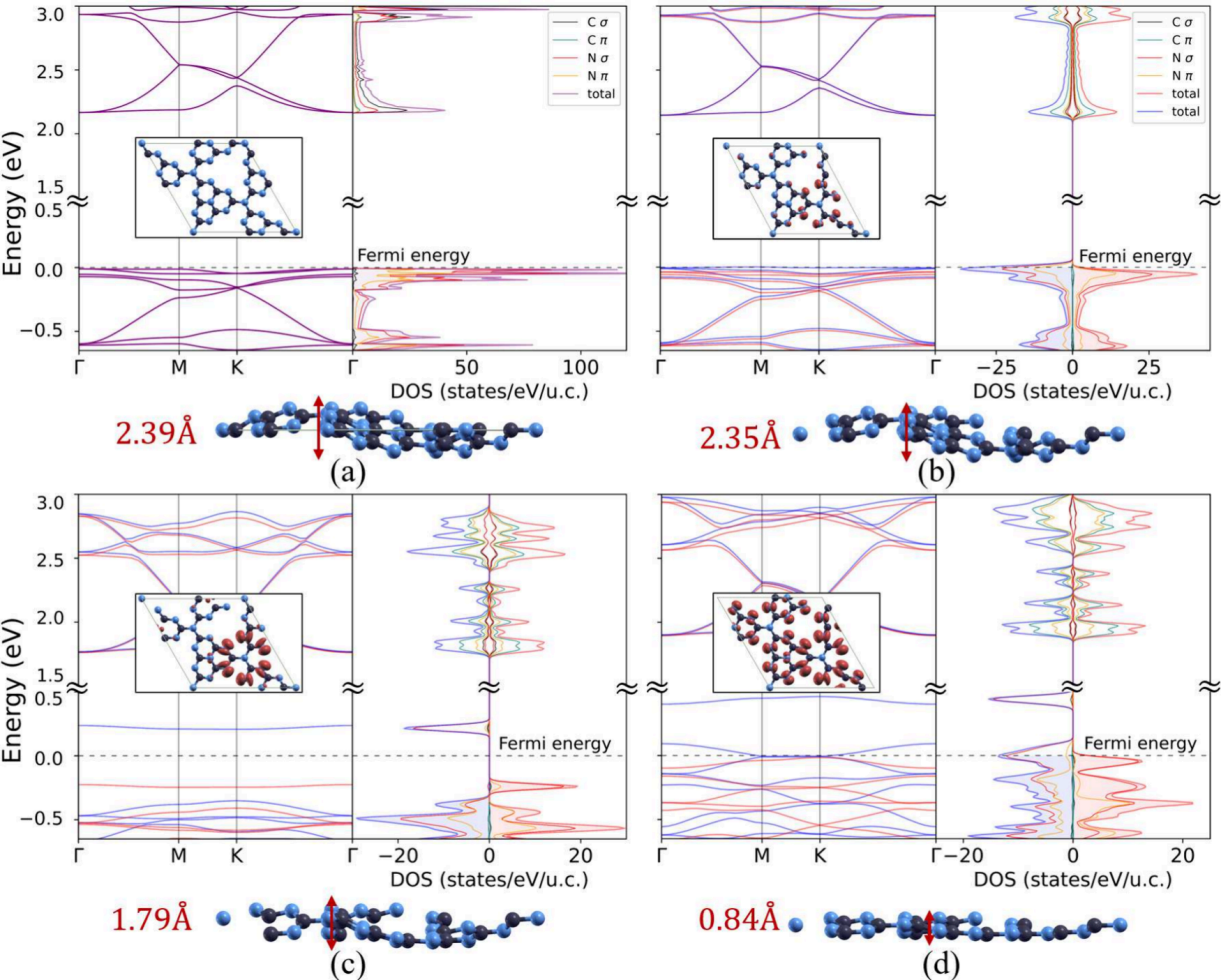
Electronic structure and corrugation of single-layer g-C_3_N_4_. Each panel represents the electronic structure, the density of states, and the corrugation as a function of field-effect doping: (a) undoped, (b) *n*_h_ = 1.66 × 10^13^ holes/cm^2^ (*n*_h_ = 0.2 holes/42 atoms u.c.), (c) *n*_h_ = 8.29 × 10^13^ holes/cm^2^ (*n*_h_ = 1 hole/42 atoms u.c.), and (d) *n*_h_ = 1.658 × 10^14^ holes/cm^2^ (*n*_h_ = 2 holes/42 atoms u.c.). The dashed line marks the Fermi level *E*_F_. The insets in the electronic structures represent the top view of the structure in panel (a), while it shows the collinear magnetization density, *m*(**r**) = ρ_↑_(**r**) – ρ_↓_(**r**), in the other panels.

The electronic structure of single-layer undoped g-C_3_N_4_ is shown in Figure 1(a). At the top of the valence band, two very flat degenerate bands are present, composed of nitrogen orbitals, mostly of σ character (see section S2 of the Supporting Information for a plot of the modulus of the Bloch functions at zone center for the two states at the top of the valence band). The real space localization of the two highest energy N lone-pair states generates a sharp peak in the density of states and a small electron kinetic energy. Finally, as the system is non-magnetic, the up and down states are degenerate.

We then model the field-effect geometry and charging of the single layer by using the method developed in refs 39 and 40 (more details are given in section S1 of the Supporting Information). We consider hole charging of g-C_3_N_4_ up to a value of *n*_h_ ≈ 2 × 10^14^ holes/cm^2^, and we perform structural optimization in field-effect geometry starting from the √3 × √3*R*30° cell. We find that, introducing of holes in the lone pairs and accounting for collinear magnetism in the structural optimization, the lattice parameter increases and the corrugation of the g-C_3_N_4_ monolayer is reduced, as shown in Figures 1 and 2. This is expected, as the smaller negative charges hosted in the lone pairs reduce their mutual Coulomb repulsion and, thus, the corrugation.

**Figure 2:**
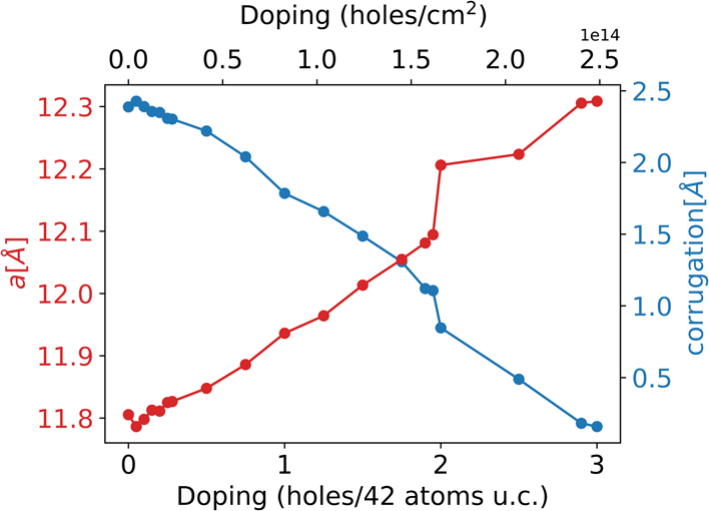
Lattice parameter (*a*) and corrugation as a function of the field-effect charging. The amount of corrugation is quantified by evaluating the maximal modulus of the difference between the *z* coordinates of the atoms.

We now investigate the ground-state properties of gated g-C_3_N_4_ with respect to the possible occurrence of superconducting and magnetic states. It is crucial to remark that, as soon as a minimal fraction of holes is added to the g-C_3_N_4_ monolayer, the ground state becomes ferromagnetic and, most interestingly, half-metallic (we label half-metal a system in which the carriers belong only or to a great majority to one spin channel). We tested for other possible magnetic orderings compatible with the √3 × √3*R*30° cell, such as antiferromagnetic ones, but we find that the lowest energy structure is always ferromagnetic. We also tested the effects of Hubbard interactions within the DFT + U formalism, but we did not find qualitative differences. Finally, we computed the orbital magnetization with the method developed in ref 41, and we found it to be of the order of 10^–3^ μ_B_ at *n*_h_ = 8.29 × 10^13^ holes/cm^2^ and, thus, negligible.

The cell magnetization as a function of doping is shown in Figure 3: it is directly proportional to the hole concentration up to a value of *n*_h_ = 8.29 × 10^13^ holes/cm^2^. The magnetization of the single layer per 42 atoms cell is sizable as it reaches the value of μ_B_, namely, approximately 0.04 μ_B_ per nitrogen, at this charging level. The associated magnetization density *m*(**r**) = ρ_↑_(**r**) – ρ_↓_(**r**), where ρ_σ_(**r**) is the spin-resolved charge density, is shown in the insets in panels (b), (c), and (d) of Figure 1 at the respective doping levels of *n*_h_ = 1.66 × 10^13^, 8.29 × 10^13^, and 1.66 × 10^14^ holes/cm^2^. The character of the magnetization density is also shown in greater detail in Figure 3: in the phase diagram, we summarize the evolution of the layer magnetization, the magnetization density, and the nature of the magnetic phase (half-metallic or insulating).

**Figure 3:**
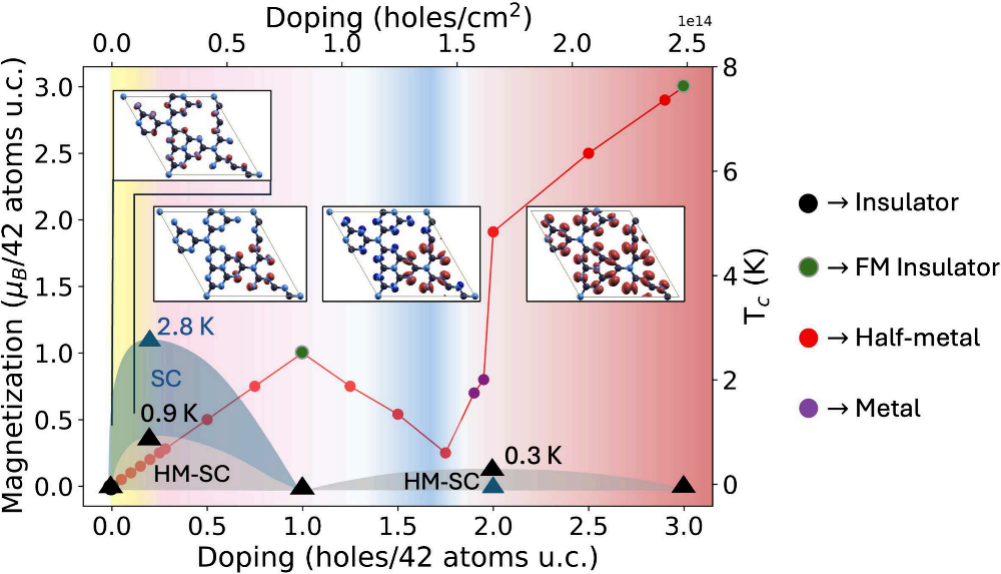
Phase diagram of hole-doped g-C_3_N_4_. We report the magnetization of the 42 atoms u.c. as a function of doping. The color of the dots labels the electronic state of the layer. The background colors correspond to the different character of the magnetization density, *m*(**r**) = ρ_↑_(**r**) – ρ_↓_(**r**), in order: in yellow, the doping region where the magnetization density has the same character of the top of the valence band of the undoped phase; in pink, the region in which the magnetization density is localized on the lone-pair-bearing atoms closer to the gate; in the blue region, some majority spin states localized on other atoms start to get depopulated; and in the red region, only minority spin states are depopulated and the spins are spread out on the whole layer. Black (blue) triangles label the calculated superconducting *T*_c_ at the doping levels of *n*_h_ = 1.66 × 10^13^ and 1.66 × 10^14^ holes/cm^2^ in the ferromagnetic half-metallic and non-magnetic states. At *n*_h_ = 1.66 × 10^14^ holes/cm^2^, the paramagnetic solution is insulating. Shaded areas are guides for the eye.

At very low doping, the magnetization density is due to one of the two top-valence states that are degenerate in the absence of gating (the electric field breaks the degeneracy, as seen by comparing section S2 of the Supporting Information to Figure 1(a)). With the increase of the doping, the electric field strongly affects the corrugation and the electronic structure; thus, a complex interplay between the highest occupied bands takes place, giving rise to the change in character of the magnetization density that is reported in Figure 3 for *n*_h_ ≈ 1.24 × 10^13^ holes/cm^2^.

The complex behavior in this doping range is mirrored in the electronic structure in panels (b) and (c) of Figure 1. Indeed, the electric field splits the two degenerate top valence bands, changing the character of the highest occupied one and pushing the other at low energy far below the gap. The band at the Fermi level is split in two by magnetism and generates a truly half-metallic state with tunable magnetization.

At *n*_h_ = 8.29 × 10^13^ holes/cm^2^, corresponding to the filling of one hole per 42 atoms cell, the highest energy minority spin lone-pair band is completely empty as opposed to the majority one, which is completely filled, with a gap existing between the two (see Figure 1(c)). Thus, field-effect doping of pristine g-C_3_N_4_ leads first to a magnetic half-metallic state and then, at the first integer hole filling, to a ferromagnetic band insulating state.

The linear behavior of the magnetization versus charging is shown in Figure 3, showcasing the advantage and versatility of field-effect doping of pristine g-C_3_N_4_ with respect to the previously proposed carbon–nitrogen chemical substitution leading to g-C_4_N_3_.

At intermediate charging, between *n*_h_ = 8.29 × 10^13^ holes/cm^2^ and *n*_h_ = 1.66 × 10^14^ holes/cm^2^, the behavior of the magnetization is more complex, with a decrease in the total magnetization.

For levels higher than *n*_h_ = 1.66 × 10^14^ holes/cm^2^, the magnetization density spreads over the whole monolayer, with all of the nitrogen lone pairs contributing to it. This is due to both structural and electronic effects: as reported in Figure 2, the layer flattens out as we dope it, leading to a larger σ character of the lone-pair states and to a partial recovery of the ideal flat case; furthermore, at large doping levels, we dope more than one band, corresponding to states localized on different lone pairs.

The linear regime of the magnetization is recovered at larger doping values: this behavior is retained up to the highest doping level considered of *n*_h_ = 2.49 × 10^14^ holes/cm^2^ (3 holes/42 atoms u.c.), where the electronic structure is once again that of an insulator at an odd-integer hole-doping value.

As happens in other carbon-based 2D materials, the magnetic order may compete or coexist with a superconducting state. For this reason, we calculate the superconducting properties at the two hole densities *n*_h_ = 1.66 × 10^13^ and 1.66 × 10^14^ holes/cm^2^ (see section S5 of the Supporting Information for more details). We consider both a paramagnetic superconducting state (competition) and a ferromagnetic half-metallic superconducting state (coexistence).

We find a moderate electron–phonon coupling (EPC) in both cases, with the values reported in Table 1. In the half-metallic state (coexistence), we obtain the non-negligible *T*_c_ values of 0.9 K and 0.3 K at the two hole densities of *n*_h_ = 1.66 × 10^13^ and 1.66 × 10^14^ holes/cm^2^, respectively. These values are of the same order as the *T*_c_ measured in TBG and are substantially larger than the 100 mK *T*_c_ of rhombohedral trilayer graphene. In the paramagnetic state (competition), the EPC is substantially larger, as shown in Table 1, leading to an enhanced *T*_c_ = 2.8 K for *n*_h_ = 1.66 × 10^13^ holes/cm^2^. This is easily explained by the fact that, at *n*_h_ = 1.66 × 10^13^ holes/cm^2^, the electronic DOS at the Fermi level is higher in the non-magnetic state than in the magnetic one. At the higher doping of *n*_h_ = 1.66 × 10^14^ holes/cm^2^, the material enters a band insulating state in the absence of spin polarization, leading to a suppression of superconductivity. The results of the *T*_c_ calculations are illustrated graphically in Figure 3.

**Table 1:** Magnetic State (HM, Half-Metallic; PM, Paramagnetic), Electron–Phonon Coupling, Logarithmic Average of the Phonon Frequencies, and Critical Temperature for the Investigated Doping Levels

*n*_h_ (holes/cm^2^)	state	λ	ω_log_ (meV)	*T*_c_ (K)
1.66 × 10^13^	HM	0.37	27.8	0.9
	PM	0.46	28.7	2.8
1.66 × 10^14^	HM	0.31	33.8	0.3

In this work, we showed that the depletion of nitrogen lone-pair states in g-C_3_N_4_ generates ultraflat bands at the Fermi level and leads to correlated states. By using first-principles electronic structure calculations in field-effect configuration, we demonstrated that a rich phase diagram occurs. At low hole concentrations, a half-metallic state emerges with tunable magnetization increasing linearly with carrier density and reaching up to 1 μ_B_/3 f.u. Such extreme tunability of the half-metallic state is very promising for spintronics.^42^ At these doping levels, an interplay between the half-metallic and superconducting phases takes place, possibly leading to phonon-mediated unconventional pairing. At larger hole charging, a succession of ferromagnetic insulating, metallic, and half-magnetic phases occurs, ultimately leading, at a filling of 3 holes per cell, to a second ferromagnetic insulating state.

Finally, our work identifies the depletion of nitrogen lone pairs as a new platform for generating ultraflat bands hosting correlated states. As lone-pair states are generally localized in real space and occur in several compounds, ranging from single-layer Bi^43^ to few-layer metal halides,^44^ our strategy for the formation of ultraflat band systems is general and easily portable to other compounds.

## Supplementary Material





## Notes

Technical details, crystal structures, extra band structures, phonon dispersions, electron doping, and superconducting properties (PDF)
Transparent Peer Review report available (PDF)
