# Supplementary material for: Interplay of Superconductivity, Ferromagnetism, and Half-Metallicity in Gated Single-Layer g‑C3N4
Source: J Phys Chem Lett. 2025 Jun 3;16(23):5739–44. doi: 10.1021/acs.jpclett.5c01013 (PMC12169656; doi:10.1021/acs.jpclett.5c01013)
Supplement: Supplementary file 1 [file jz5c01013_si_001.pdf]

Supporting Information – Supporting Information for "Interplay of Superconductivity, Ferromagnetism and Half-Metallicity in Gated Single-Layer g-C_3N_4" .

Pietro Nicolò Brangi,* Francesca Martini, Pierluigi Cudazzo, and Matteo Calandra*

Department of Physics, University of Trento, Via Sommarive 14, 38123 Povo, Italy

E-mail: pietronicolo.brangi@unitn.it; m.calandrabuonaura@unitn.it

S1 Technical details and crystal structures

S1.1 Technical details

We study the effects of doping in the most stable heptazine phase of g-C_3N_4. The calculations are carried out with the Density Functional Theory (DFT)[1,2] formalism using the QUANTUM ESPRESSO code v5.1[3,4] . We employ the PBE exchange-correlation functional[5] and the pseudopotentials are taken from the SSSP library of Materials Cloud[6] (v.1.3.0 - precision; N: uspp;[7] C: PAW pseudopotential).

FET doping Doping is introduced in a Field Effect Transistor (FET) configuration using the method developed and extensively described in Refs.[8,9] : we model the gate of the FET as a charged plate acting as an external potential for the DFT calculation, while the Coulomb interaction is cut off in the direction perpendicular to the gate to avoid spurious interactions between different images. Furthermore, a potential barrier is introduced between the gate and the charged bidimensional material to simulate the dielectric which has both an insulating and a repulsive effect (i.e.: it doesn't allow the charge to spill from the layer to the gate and it prevents the layer to collapse into the gate).

S1.2 Lattice relaxation and crystal structures

We carried out a full optimization of the cell and the atomic positions in the undoped phase and at all doping levels, with a convergence threshold of 10^{-4} Ry for the total energy and 10^{-3}Ry/Bohr for the forces acting on the atoms. The total energy convergence threshold for single point self-consistent calculations was set to 10^{-10} Ry for the lattice relaxations, in order to obtain accurate forces, and 10^{-9} Ry for the band structure calculations. The plane-waves energy cutoff was set to 100 Ry for the wavefunction and to 800 Ry for the charge density, we employed a $4 \times 4 \times 1$ reciprocal space mesh for the lattice relaxations and a $6 \times 6 \times 1$ mesh for the self consistent calculations. In the undoped case, the procedure was performed for the 1×1 flat unit cell and for the 1×1, 2×2 and $\sqrt{3} \times \sqrt{3}R30°$ corrugated cells (i.e. the optimization of the internal coordinates was allowed in the direction perpendicular to the layer and some atoms were displaced to induce a corrugation), analogously to what is done in Ref.[10] . We find that the ground state of the system is the $\sqrt{3} \times \sqrt{3}R30°$ cell, with a remarkable effect of the corrugation bringing the layer thickness to 2.388 Å. The optimized structure is also dynamically stable unlike the flat one, as reported in Sec. S3. From now on we'll then refer to the $\sqrt{3} \times \sqrt{3}R30°$ supercell as the unit cell (u.c.) for corrugated g-C_3N_4. As the periodicity increases and the unit cell is enlarged (and rotated), the Brillouin zone then shrinks and rotates accordingly as reported in Fig.S1.

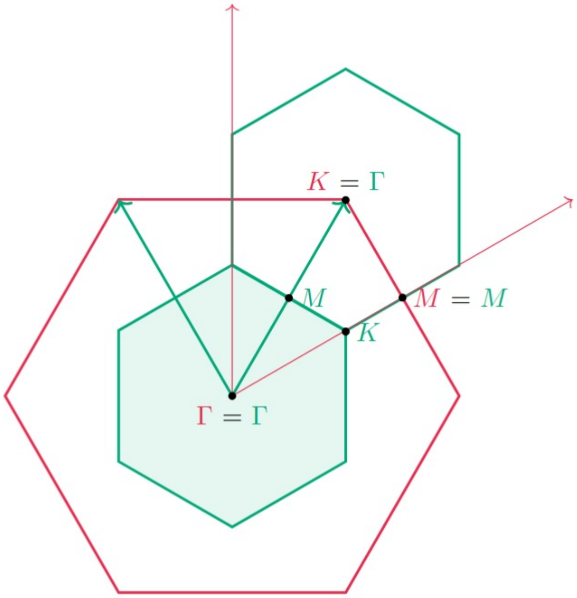

Fig. S1: Brillouin zone (BZ) of g-C_3N_4 corresponding to the 1×1 unit cell (red) and to the $\sqrt{3} \times \sqrt{3}R30°$ supercell (green). One can see that the Γ point of the 2 BZ of the supercell corresponds to the K point of the 1×1 cell's 1 BZ.

The full cell optimization in the FET configuration requires a more delicate procedure: the charged layer is attracted by the gate and it moves in that direction, until the repulsion given by the potential barrier for the electrons stops it. At the same time the atomic positions are moved and the cell changes volume. Moreover, as we are investigating magnetic

phases, we have the further degree of freedom of spin polarization in the self-consistent DFT calculations.

In these cases the structural optimization was performed first without spin polarization as the main effect driving the layer reconstruction is the depopulation of the lone pair states, which leads to a flattening of the layer. Nevertheless, the effect of spin polarization on the atomic structure is noticeable: we calculated the phonon frequencies at Γ for the doping levels of 2.32×10^{13}holes/cm^2 (1 hole/u.c.) and 4.64×10^{13}holes/cm^2 (2 holes/u.c.) for the structures optimized without allowing for spin polarization and they turn out to be dynamically unstable. The instability was cured by allowing for a further reconstruction accounting for the magnetization of the layer, as reported in Fig.S7. Thus we optimized the structure at each doping level also considering the effect of the spin polarization. The relaxed in-plane lattice parameter is reported in Tab.S1, the atomic positions are reported at the end of this file.

Tab. S1: Lattice parameters of FET-doped g-C_3N_4 at the investigated doping levels.

Doping (holes/cm^2)	a (Å)	corrugation (Å)
0	11.805	2.388
4.14×10^{12}	11.786	2.428
8.29×10^{12}	11.798	2.39
1.24×10^{13}	11.813	2.356
1.66×10^{13}	11.811	2.349
2.07×10^{13}	11.824	2.308
2.32×10^{13}	11.827	2.303
4.14×10^{13}	11.848	2.220
6.21×10^{13}	11.886	2.040
8.29×10^{13}	11.936	1.785
1.04×10^{14}	11.964	1.658
1.24×10^{14}	12.013	1.487
1.45×10^{14}	12.055	1.308
1.57×10^{14}	12.081	1.121
1.61×10^{14}	12.094	1.107
1.66×10^{14}	12.206	0.847
2.07×10^{14}	12.223	0.488
2.40×10^{14}	12.305	0.181
2.48×10^{14}	12.309	0.158

S2 Low and medium doping behaviour

S2.1 Low doping band structures

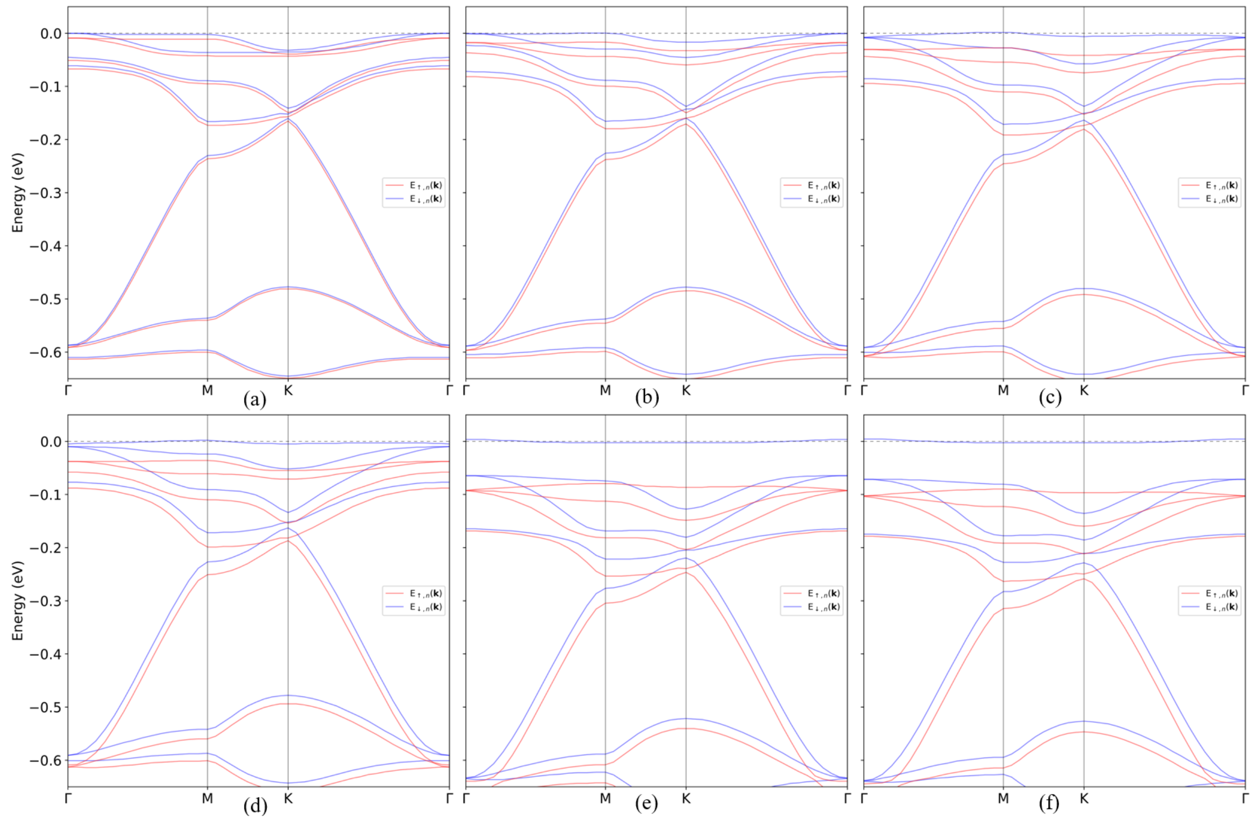

Fig. S2: Electronic band structure of field-effect doped g-C_3N_4 at the doping levels of (a) 4.14×10^{12}holes/cm^2 (0.05 holes/u.c.), (b) 8.29×10^{12}holes/cm^2 (0.1 holes/u.c.), (c) 1.24×10^{13}holes/cm^2 (0.15 holes/u.c.), (d) 1.66×10^{13}holes/cm^2 (0.2 holes/u.c.), (e) 2.07×10^{13}holes/cm^2 (0.25 holes/u.c.), (f) 2.32×10^{13}holes/cm^2 (0.28 holes/u.c.) .

At low doping levels the spin polarized charge density changes character at the doping level $n_h = 1.24 \times 10^{13}$holes/cm^2 (0.15holes/u.c.), as highlighted in the phase diagram reported in the main paper (see Fig.3 of the main article). Here we highlight the possible reason for it. The first and the second electronic bands are degenerate for the undoped structure at Γ. While the degeneracy is lifted with the introduction of the perturbation (the electric field in the FET setup), the second and third bands become quasi-degenerate from a doping level of 0.15holes/u.c., then retaining the quasi-degeneracy for low doping values. This complex interplay and mixing between different states very close in energy leads to the behaviour highlighted in the phase diagram, with the highest occupied band changing character around the doping level of 1.24×10^{13}holes/cm^2.

S2.2 Low doping wavefunction character

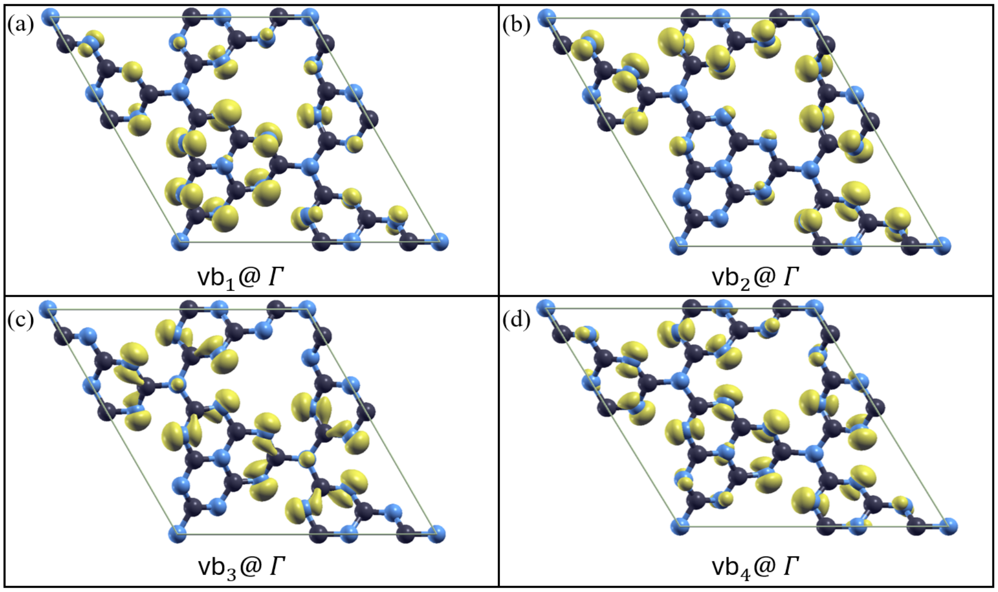

Fig. S3: Isosurfaces of the square modulus of the wavefunction's periodic part for the top 4 states of the valence band in undoped g-C_3N_4 (numbered in descending order in energy).

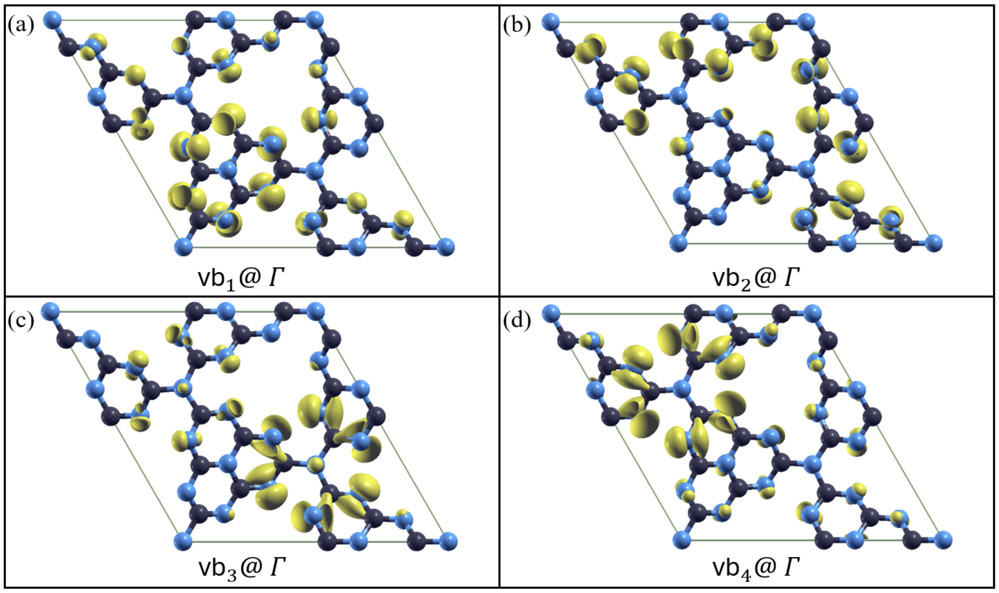

Fig. S4: Isosurfaces of the square modulus of the wavefunction's periodic part for the top 4 states of the valence band in g-C_3N_4 doped with 8.29×10^{12}holes/cm^2 (0.1holes/u.c.) (numbered in descending order in energy).

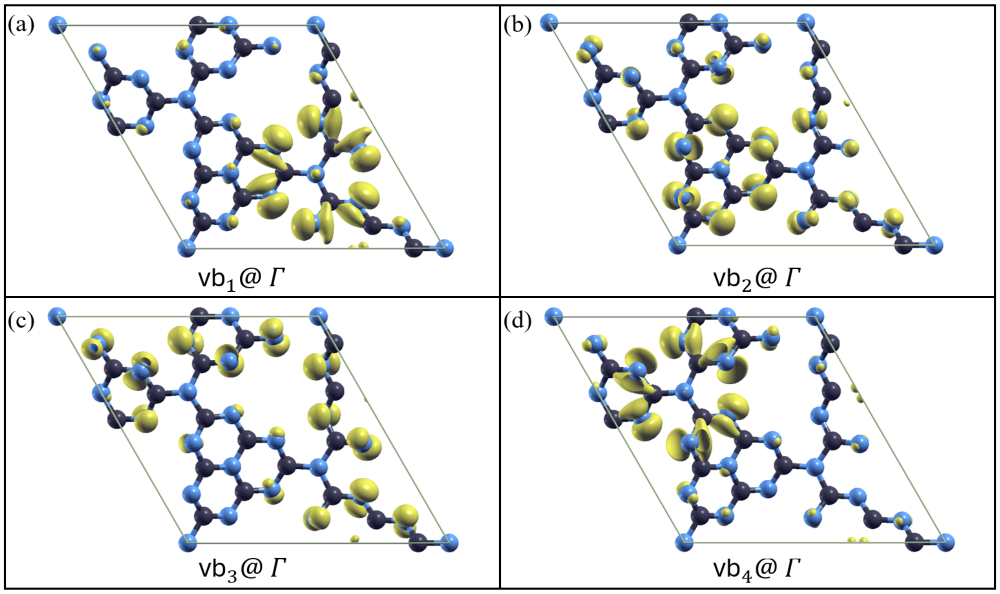

Fig. S5: Isosurfaces of the square modulus of the wavefunction's periodic part for the top 4 states of the valence band in g-C_3N_4 doped with 2.07×10^{13}holes/cm^2 (0.25holes/u.c.) (numbered in descending order in energy).

S2.3 Band structures of ferrimagnetic states

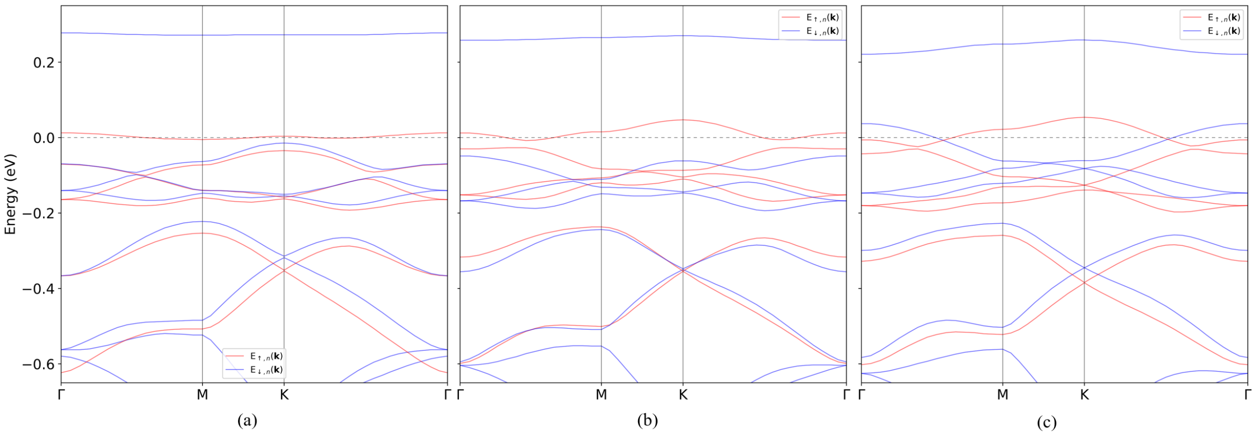

Fig. S6: Electronic band structure of field-effect doped g-C_3N_4 at the doping levels of (a) 1.24×10^{14}holes/cm^2 (1.5 holes/u.c.), (b) 1.45×10^{14}holes/cm^2 (1.75 holes/u.c.), (c) 1.57×10^{14}holes/cm^2 (1.9 holes/u.c.).

One can see in Fig.S6 (a) and (b) that only one spin channel is crossed by the Fermi level, making these states half-metallic. Nevertheless, the difference in character and in occupation between the first fully unoccupied band and the one of opposite spin character at the Fermi level causes these states to be ferrimagnetic too, since spins of opposite signs are present on different atomic sites. At higher doping (1.57×10^{14}holes/cm^2) both spin channels are present at the Fermi level making this a simple metal (see Fig.S6 (c)).

S3 Phonon dispersion relations

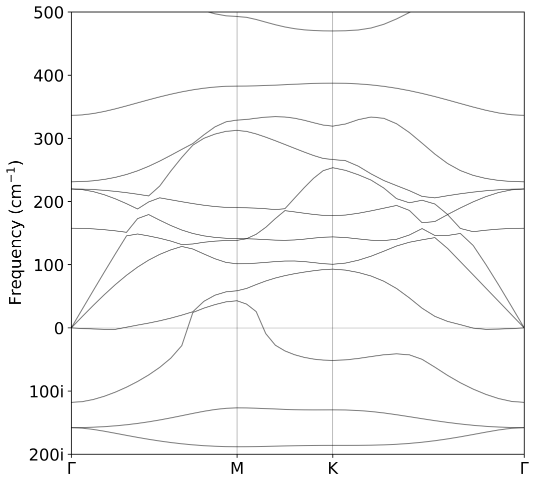

(a) Phonon dispersion relation of the flat 1×1 unit cell of undoped g-C_3N_4.

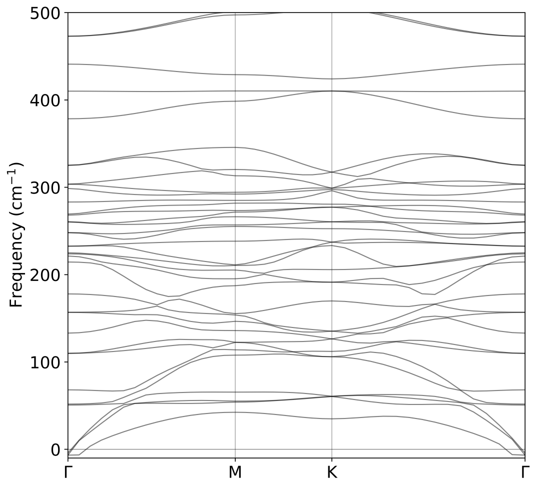

(b) Phonon dispersion relation of the corrugated $\sqrt{3} \times \sqrt{3}R30°$ unit cell of undoped g-C_3N_4.

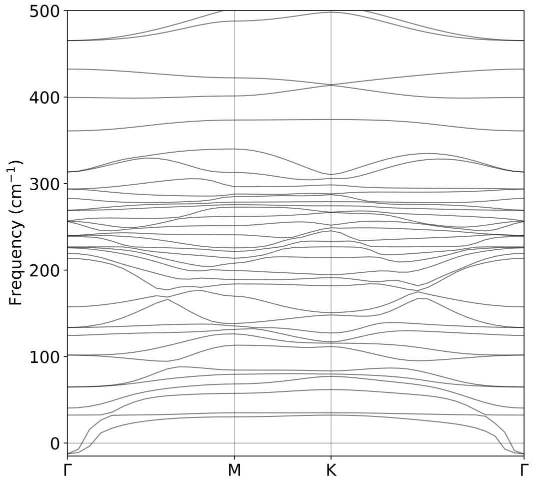

(c) Phonon dispersion relation of the corrugated $\sqrt{3} \times \sqrt{3}R30°$ unit cell of g-C_3N_4 doped with $n_h = 8.29 \times 10^{13}$holes/cm^2 (1 hole/u.c.).

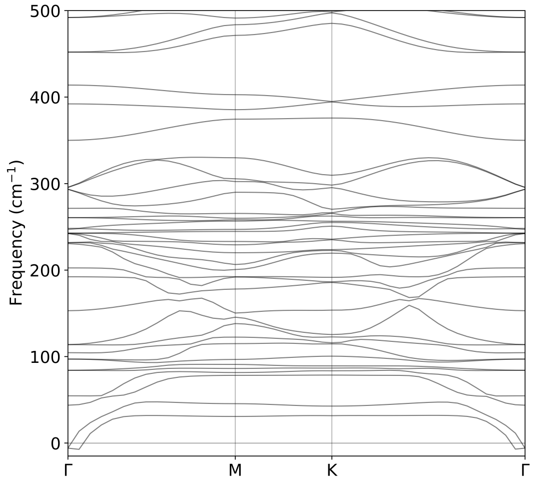

(d) Phonon dispersion relation of the corrugated $\sqrt{3} \times \sqrt{3}R30°$ unit cell of g-C_3N_4 doped with $n_h = 1.658 \times 10^{14}$holes/cm^2 (2 holes/u.c.).

Fig. S7

S4 Electron doping

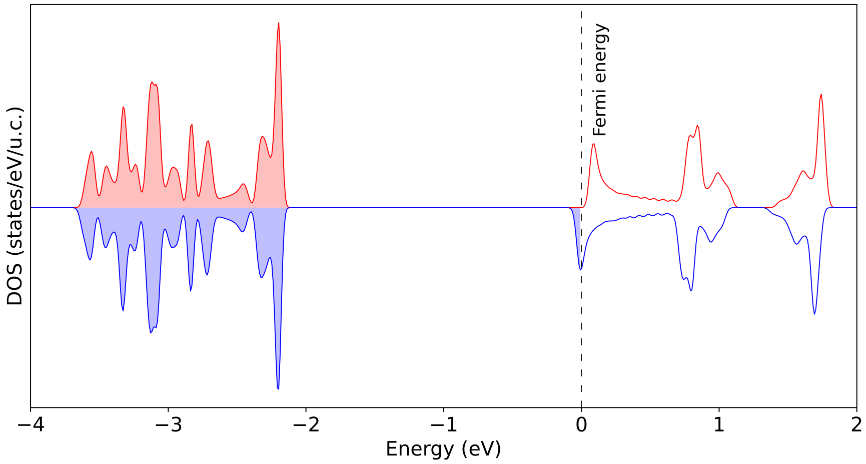

Fig. S8: Density of states of g-C_3N_4 doped in FET configuration with 0.5 electrons/u.c.

We highlight here that electron doping also leads to the stabilization of a half metallic state even for a relatively small amount of doping. This is of crucial importance for the applications in the field of photo-catalytic cells, because both hole and electron doping - which may be induced by the electric field in the cell - lead to the stabilization of a half metallic state.

S5 Superconducting state

We carried out calculations of the electron phonon coupling (EPC) and superconducting critical temperature T_c using the McMillan formula. Here we report the Eliashberg functions $\alpha^2F(\omega) = \frac{1}{2N_q}\sum_{\mathbf{q}\nu}\lambda_{\mathbf{q}\nu}\omega_{\mathbf{q}\nu}\delta(\omega-\omega_{\mathbf{qu}\nu})$ and the value of the average electron phonon coupling $\lambda(\omega) = 2\int_0^\omega d\omega'\alpha^2F(\omega')/\omega'$ at the two doping levels $n_h = 1.66\times10^{13}$ holes/cm^2 and $n_h = 1.66\times10^{14}$ holes/cm^2. The critical temperatures are calculated with McMillan's formula $T_c = \frac{\langle\omega\rangle_{log}}{1.2}\exp[-\frac{1.04(1+\lambda)}{\lambda-\mu^*(1+0.62\lambda)}]$, where $\langle\omega\rangle_{log}$ =exp$[\frac{2}{\lambda}\int_0^{+\infty}\alpha^2F(\omega)\log(\omega)/\omega d\omega]$ is the phonon frequencies logarithmic average and μ^* is the screened Coulomb pseudopotential, which we assume to be $\mu^* = 0.1$. The values of the $\mathbf{q}$ and mode specific EPC $\lambda_{\mathbf{q}\nu}$ used in the calculation of $\alpha^2F(\omega)$ are converged with respect to the smearing in each of the three cases reported: (i) $n_h = 1.66\times10^{13}$ holes/cm^2 in the ferromagnetic half-metallic phase, (ii) $n_h = 1.66\times10^{13}$ holes/cm^2 in the non-magnetic phase and (iii) $n_h = 1.66\times10^{14}$ holes/cm^2 in the ferromagnetic half-metallic phase.

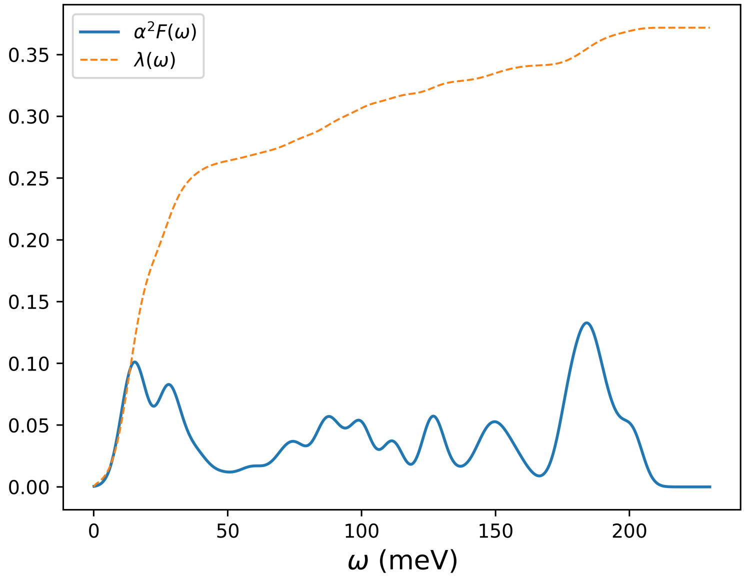

Fig. S9: Eliashberg function and average electron-phonon coupling as a function of the phonon frequency, in g-C_3N_4 hole doped with $n_h = 1.66\times10^{13}$ holes/cm^2, in its ferromagnetic half-metallic phase.

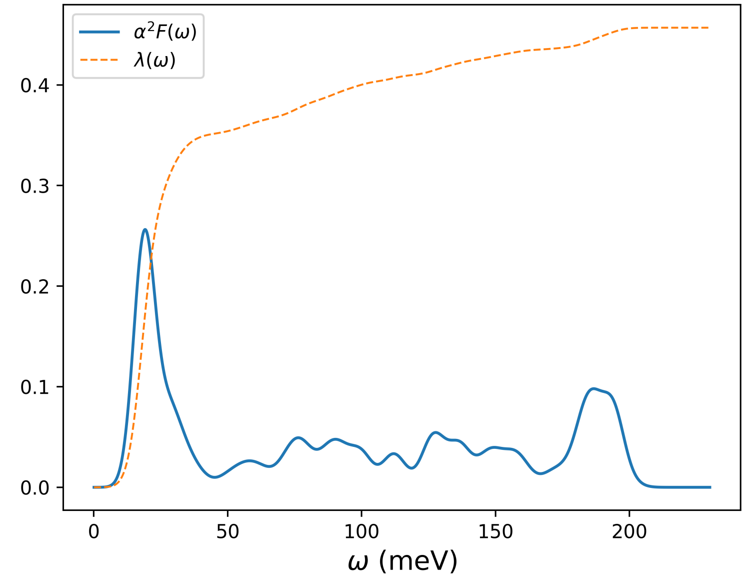

Fig. S10: Eliashberg function and average electron-phonon coupling as a function of the phonon frequency, in g-C_3N_4 hole doped with $n_h = 1.66 \times 10^{13}$ holes/cm^2, in its non magnetic phase.

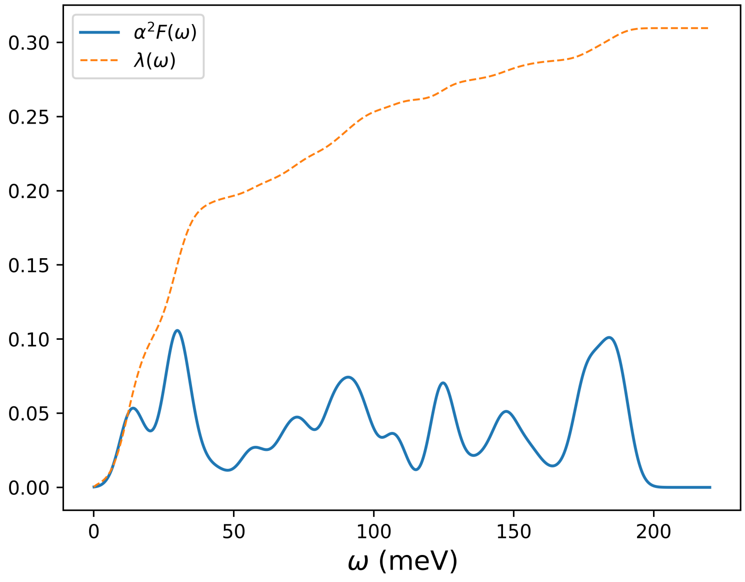

Fig. S11: Eliashberg function and average electron-phonon coupling as a function of the phonon frequency, in g-C_3N_4 hole doped with $n_h = 1.66{\times}10^{14}$ holes/cm^2, in its ferromagnetic half-metallic phase.

S6 Inadequacy of uniform background doping

Here we address the effect of the uniform field acting on the g-C_3N_4 layer in a FET setup. Such field is also commonly referred to as displacement field. In our work we include both the effect of charging and the effect of the displacement field using the Field Effect Transistor (FET) setup, as reported in Ref.[9] . Doping is not performed through a homogeneous compensating background; rather, it is taken into account by introducing the displacement field potential as an external potential in the Kohn-Sham Hamiltonian. This allows us to simulate a FET-like experimental setup in which the effect of the field is crucial (see for example Ref.[11,12]).

To disentangle the effect of the displacement field and the effect of charging, we computed the properties of the monolayer of g-C_3N_4 when doped with uniform background doping. In Fig. S12 we report the band structure and magnetization density of the layer uniformly doped with n_h = 1 hole/ 42 atoms u.c.. Comparing this with the band structure of g-C_3N_4 at the same doping level but with a FET setup (see Fig.1 (c) of the main paper) one can notice the remarkable effect of the displacement field. When uniform doping is employed (i) the electronic structure is metallic as opposed to insulating, (ii) the magnetization density is spread over many lone pairs (according to the character of the upper valence band) and (iii) the upper valence band is not split from the second one.

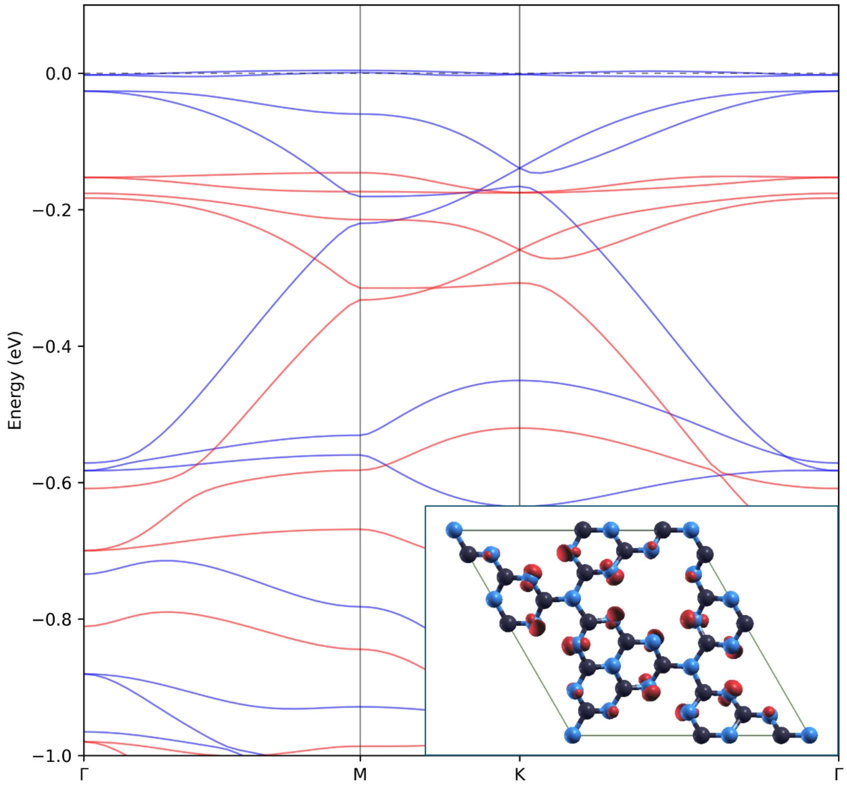

Fig. S12: Spin polarized band structure of g-C_3N_4 uniformly doped with $n_h = 8.29 \times 10^{13}$ holes/cm^2 (n_h = 1 hole/ 42 atoms u.c.). In the inset the collinear magnetization density, $m(\mathbf{r}) = \rho_\uparrow(\mathbf{r}) - \rho_\downarrow(\mathbf{r})$.

The different behaviour can be explained in terms of simple electrostatics: the negatively

charged gate attracts the positive charges introduced with doping, causing a change in the localization of the holes (which are found on the nitrogen lone pairs closer to the gate) which is also reflected in a modification of the wavefunction and magnetization characters.

Atomic positions

- Undoped structure

ATOMIC_POSITIONS crystal
N 0.0000000000 0.0000000000 0.0000000000
C 0.0000000000 0.5440224037 0.0000000000
N 0.0000000000 0.6618576696 0.0000000000
C -0.0000000000 0.8805117483 0.0000000000
C 0.5440224037 1.0000000000 0.0000000000
N 0.6618576696 1.0000000000 0.0000000000
C 0.8805117483 1.0000000000 0.0000000000
N 0.0939724467 0.8791701124 0.0261123787
C 0.1005867307 0.7699103820 0.0237940799
N 0.1127657127 0.5475221957 0.0078953115
N 0.1208298876 0.2148023345 0.0261123787
C 0.1194882517 0.1194882517 0.0000000000
N 0.2148023345 0.1208298876 -0.0261123787
C 0.2300896180 0.3306763487 0.0237940799
N 0.2404982547 0.4394342106 0.0424924157
C 0.2077667886 0.6568210648 0.0273519611
N 0.1989359558 0.7595017453 0.0424924157
N 0.3333333000 0.6666667000 0.0315753457
C 0.3431789352 0.5509458239 0.0273519611
N 0.3381423304 0.3381423304 0.0000000000
C 0.3306763487 0.2300896180 -0.0237940799
N 0.4394342106 0.2404982547 -0.0424924157
C 0.4559775963 0.4559775963 0.0000000000
N 0.4524778043 0.5652436172 0.0078953115
C 0.4490541761 0.7922332114 0.0273519611
N 0.4347563828 0.8872342873 0.0078953115
N 0.5605657894 0.8010640442 0.0424924157
N 0.5652436172 0.4524778043 -0.0078953115
C 0.5509458239 0.3431789352 -0.0273519611
N 0.5475221957 0.1127657127 -0.0078953115
C 0.6568210648 0.2077667886 -0.0273519611
N 0.6666667000 0.3333333000 -0.0315753457
C 0.6693236513 0.8994132693 0.0237940799
N 0.7851976655 0.9060275533 0.0261123787
N 0.8010640442 0.5605657894 -0.0424924157
C 0.7922332114 0.4490541761 -0.0273519611
N 0.7595017453 0.1989359558 -0.0424924157
C 0.7699103820 0.1005867307 -0.0237940799
N 0.8791701124 0.0939724467 -0.0261123787
N 0.8872342873 0.4347563828 -0.0078953115

C 0.8994132693 0.6693236513 -0.0237940799
N 0.9060275533 0.7851976655 -0.0261123787

- 4.14×10^{12} holes/cm^2

  ATOMIC_POSITIONS crystal
  N 0.000000000 0.000000000 0.000044977
  C 0.000078564 0.543500093 -0.000068406
  N 0.000146938 0.661589528 -0.000103460
  C -0.000022973 0.880449079 0.000013591
  C 0.543421529 0.999921436 -0.000068406
  N 0.661442590 0.999853062 -0.000103460
  C 0.880472052 1.000022973 0.000013591
  N 0.093413663 0.878796430 0.028596752
  C 0.100119551 0.769421286 0.026051905
  N 0.112704660 0.546808240 0.009134151
  N 0.121203570 0.214617232 0.028596752
  C 0.119550921 0.119527948 0.000013591
  N 0.214591946 0.121111354 -0.028532527
  C 0.230578714 0.330698266 0.026051905
  N 0.241147921 0.439264888 0.046666248
  C 0.207367786 0.656184163 0.030388537
  N 0.198116967 0.758852079 0.046666248
  N 0.333333300 0.666666700 0.035227573
  C 0.343815837 0.551183723 0.030388537
  N 0.338410472 0.338557410 -0.000103460
  C 0.330627477 0.230565392 -0.026102597
  N 0.439251023 0.241001503 -0.046583221
  C 0.456499907 0.456578471 -0.000068406
  N 0.453191760 0.565896521 0.009134151
  C 0.448816277 0.792632214 0.030388537
  N 0.434103479 0.887295340 0.009134151
  N 0.560735112 0.801883033 0.046666248
  N 0.565806605 0.453213202 -0.009177469
  C 0.551132569 0.343762247 -0.030376673
  N 0.546786798 0.112593302 -0.009177469
  C 0.656237753 0.207370221 -0.030376673
  N 0.666666700 0.333333300 -0.035127921
  C 0.669301734 0.899880449 0.026051905
  N 0.785382768 0.906586337 0.028596752
  N 0.801750480 0.560748977 -0.046583221
  C 0.792629779 0.448867431 -0.030376673
  N 0.758998497 0.198249520 -0.046583221

C 0.769434608 0.100062086 -0.026102597
N 0.878888646 0.093480592 -0.028532527
N 0.887406698 0.434193395 -0.009177469
C 0.899937914 0.669372523 -0.026102597
N 0.906519408 0.785408054 -0.028532527

- 8.29×10^{12} holes/cm^2

ATOMIC_POSITIONS crystal
N 0.000000000 0.000000000 -0.057822699
C 0.000463007 0.544049864 -0.058533824
N 0.000381478 0.661946505 -0.058309008
C -0.000066498 0.880530673 -0.057897288
C 0.543586855 0.999536993 -0.058533824
N 0.661565026 0.999618522 -0.058309008
C 0.880597171 1.000066498 -0.057897288
N 0.093848974 0.878993417 -0.029625587
C 0.100524178 0.769728033 -0.032298146
N 0.113163175 0.547460912 -0.049641759
N 0.121006583 0.214855557 -0.029625587
C 0.119469327 0.119402829 -0.057897288
N 0.214694199 0.120565656 -0.086083361
C 0.230271967 0.330796148 -0.032298146
N 0.240825377 0.439488484 -0.011895671
C 0.207737835 0.656594073 -0.028307189
N 0.198663107 0.759174623 -0.011895671
N 0.333333300 0.666666700 -0.023457286
C 0.343405927 0.551143863 -0.028307189
N 0.338053495 0.338434974 -0.058309008
C 0.330567095 0.230021526 -0.083734625
N 0.439242148 0.239933366 -0.103742048
C 0.455950136 0.456413145 -0.058533824
N 0.452539088 0.565702366 -0.049641759
C 0.448856137 0.792262165 -0.028307189
N 0.434297634 0.886836825 -0.049641759
N 0.560511516 0.801336893 -0.011895671
N 0.565264549 0.453225053 -0.067466878
C 0.550927529 0.343297444 -0.087980761
N 0.546774947 0.112039397 -0.067466878
C 0.656702556 0.207629984 -0.087980761
N 0.666666700 0.333333300 -0.092497451
C 0.669203852 0.899475822 -0.032298146
N 0.785144443 0.906151026 -0.029625587

N 0.800691219 0.560757852 -0.103742048
C 0.792370016 0.449072471 -0.087980761
N 0.760066634 0.199308781 -0.103742048
C 0.769978474 0.100545568 -0.083734625
N 0.879434344 0.094128543 -0.086083361
N 0.887960603 0.434735451 -0.067466878
C 0.899454432 0.669432905 -0.083734625
N 0.905871457 0.785305801 -0.086083361

- 1.24×10^{13} holes/cm^2

ATOMIC_POSITIONS crystal
N 0.000000000 0.000000000 -0.058399388
C 0.000653619 0.544640970 -0.059984375
N 0.000313000 0.662239467 -0.059427786
C -0.000280520 0.880505740 -0.058594733
C 0.543987350 0.999346381 -0.059984375
N 0.661926466 0.999687000 -0.059427786
C 0.880786259 1.000280520 -0.058594733
N 0.093964521 0.878942707 -0.030662084
C 0.100769140 0.769909260 -0.033597435
N 0.113286487 0.548042197 -0.051437033
N 0.121057293 0.215021814 -0.030662084
C 0.119494260 0.119213741 -0.058594733
N 0.214720726 0.119907176 -0.086546900
C 0.230090740 0.330859881 -0.033597435
N 0.240560227 0.439762502 -0.013594168
C 0.208020381 0.656981382 -0.030156125
N 0.199202274 0.759439773 -0.013594168
N 0.333333300 0.666666700 -0.025473296
C 0.343018618 0.551039100 -0.030156125
N 0.337760533 0.338073534 -0.059427786
C 0.330395999 0.229269603 -0.084352629
N 0.439070459 0.238886729 -0.104081769
C 0.455359030 0.456012650 -0.059984375
N 0.451957803 0.565244393 -0.051437033
C 0.448960900 0.791979619 -0.030156125
N 0.434755607 0.886713513 -0.051437033
N 0.560237498 0.800797726 -0.013594168
N 0.564595278 0.452808750 -0.068818715
C 0.550702698 0.342698349 -0.088759566
N 0.547191250 0.111786429 -0.068818715
C 0.657301651 0.208004248 -0.088759566

N 0.666666700 0.333333300 -0.093157982
C 0.669140119 0.899230860 -0.033597435
N 0.784978186 0.906035479 -0.030662084
N 0.799816270 0.560929541 -0.104081769
C 0.791995752 0.449297302 -0.088759566
N 0.761113271 0.200183730 -0.104081769
C 0.770730397 0.101126396 -0.084352629
N 0.880092824 0.094813551 -0.086546900
N 0.888213571 0.435404722 -0.068818715
C 0.898873604 0.669604001 -0.084352629
N 0.905186449 0.785279274 -0.086546900

- 1.66×10^{13} holes/cm^2

ATOMIC_POSITIONS crystal
N 0.000000000 0.000000000 -0.058438083
C 0.000686050 0.544595369 -0.060077780
N 0.000543554 0.662329484 -0.059683051
C -0.000368478 0.880486764 -0.058640647
C 0.543909318 0.999313950 -0.060077780
N 0.661785929 0.999456446 -0.059683051
C 0.880855240 1.000368478 -0.058640647
N 0.094033428 0.878973920 -0.030784674
C 0.100886922 0.769987061 -0.033804309
N 0.113068879 0.547738952 -0.051217192
N 0.121026080 0.215059508 -0.030784674
C 0.119513236 0.119144760 -0.058640647
N 0.214782259 0.119734032 -0.086498582
C 0.230012939 0.330899862 -0.033804309
N 0.240372038 0.439733775 -0.013793722
C 0.207974490 0.656886430 -0.030149195
N 0.199361736 0.759627962 -0.013793722
N 0.333333300 0.666666700 -0.025454397
C 0.343113570 0.551088161 -0.030149195
N 0.337670516 0.338214071 -0.059683051
C 0.330355190 0.229210672 -0.084410895
N 0.439102549 0.238733568 -0.104028447
C 0.455404631 0.456090682 -0.060077780
N 0.452261048 0.565330029 -0.051217192
C 0.448911839 0.792025510 -0.030149195
N 0.434669971 0.886931121 -0.051217192
N 0.560266225 0.800638264 -0.013793722
N 0.564616723 0.452881086 -0.069020739

C 0.550728931 0.342650965 -0.088852710
N 0.547118914 0.111735539 -0.069020739
C 0.657349035 0.208077865 -0.088852710
N 0.666666700 0.333333300 -0.093230186
C 0.669100138 0.899113078 -0.033804309
N 0.784940492 0.905966572 -0.030784674
N 0.799631020 0.560897451 -0.104028447
C 0.791922135 0.449271069 -0.088852710
N 0.761266432 0.200368980 -0.104028447
C 0.770789328 0.101144519 -0.084410895
N 0.880265968 0.095048228 -0.086498582
N 0.888264461 0.435383277 -0.069020739
C 0.898855481 0.669644810 -0.084410895
N 0.904951772 0.785217741 -0.086498582

- 2.07×10^{13} holes/cm^2

  ATOMIC_POSITIONS crystal
  N 0.000000000 0.000000000 -0.055267734
  C 0.001278660 0.545429591 -0.060277559
  N 0.000139651 0.662466894 -0.058483219
  C -0.001181355 0.880159106 -0.055860572
  C 0.544150929 0.998721340 -0.060277559
  N 0.662327243 0.999860349 -0.058483219
  C 0.881340459 1.001181355 -0.055860572
  N 0.093412646 0.877957744 -0.028505187
  C 0.100600946 0.769430479 -0.032442755
  N 0.113828192 0.548755007 -0.052168046
  N 0.122042256 0.215454902 -0.028505187
  C 0.119840894 0.118659541 -0.055860572
  N 0.214613233 0.118142519 -0.083637908
  C 0.230569521 0.331170469 -0.032442755
  N 0.241064595 0.440705149 -0.013261677
  C 0.208323539 0.657088664 -0.030496321
  N 0.199640553 0.758935405 -0.013261677
  N 0.333333300 0.666666700 -0.026029537
  C 0.342911336 0.551234976 -0.030496321
  N 0.337533106 0.337672757 -0.058483219
  C 0.329910603 0.227640773 -0.082306431
  N 0.438339305 0.236628260 -0.101939781
  C 0.454570409 0.455849071 -0.060277559
  N 0.451244993 0.565073286 -0.052168046
  C 0.448765024 0.791676461 -0.030496321

N 0.434926714 0.886171808 -0.052168046
N 0.559294851 0.800359447 -0.013261677
N 0.563610297 0.452788763 -0.069618233
C 0.550232587 0.341744989 -0.088053887
N 0.547211237 0.110821435 -0.069618233
C 0.658255011 0.208487496 -0.088053887
N 0.666666700 0.333333300 -0.092308635
C 0.668829531 0.899399054 -0.032442755
N 0.784545098 0.906587354 -0.028505187
N 0.798288957 0.561660695 -0.101939781
C 0.791512504 0.449767413 -0.088053887
N 0.763371740 0.201711043 -0.101939781
C 0.772359227 0.102269829 -0.082306431
N 0.881857481 0.096470715 -0.083637908
N 0.889178565 0.436389703 -0.069618233
C 0.897730171 0.670089397 -0.082306431
N 0.903529285 0.785386767 -0.083637908

- 2.32×10^{13} holes/cm^2

  ATOMIC_POSITIONS crystal
  N 0.000000000 0.000000000 -0.055652901
  C 0.001495549 0.545578005 -0.060762568
  N 0.000265048 0.662545338 -0.058855702
  C -0.001125974 0.880185079 -0.056184354
  C 0.544082455 0.998504451 -0.060762568
  N 0.662280290 0.999734952 -0.058855702
  C 0.881311052 1.001125974 -0.056184354
  N 0.093434041 0.877959761 -0.028806231
  C 0.100696763 0.769478360 -0.032772811
  N 0.113986405 0.548903250 -0.052637291
  N 0.122040239 0.215474280 -0.028806231
  C 0.119814921 0.118688948 -0.056184354
  N 0.214648878 0.118126666 -0.083883006
  C 0.230521640 0.331218403 -0.032772811
  N 0.241017129 0.440777691 -0.013622323
  C 0.208417163 0.657177990 -0.030909957
  N 0.199760561 0.758982871 -0.013622323
  N 0.333333300 0.666666700 -0.026439231
  C 0.342822010 0.551239275 -0.030909957
  N 0.337454662 0.337719710 -0.058855702
  C 0.329882596 0.227592964 -0.082553018
  N 0.438347579 0.236463651 -0.102084153

C 0.454421995 0.455917545 -0.060762568
N 0.451096750 0.565083257 -0.052637291
C 0.448760725 0.791582837 -0.030909957
N 0.434916743 0.886013595 -0.052637291
N 0.559222309 0.800239439 -0.013622323
N 0.563485826 0.452863597 -0.070109758
C 0.550245291 0.341736300 -0.088367156
N 0.547136403 0.110622130 -0.070109758
C 0.658263700 0.208508890 -0.088367156
N 0.666666700 0.333333300 -0.092556081
C 0.668781597 0.899303237 -0.032772811
N 0.784525720 0.906565959 -0.028806231
N 0.798116073 0.561652421 -0.102084153
C 0.791491110 0.449754709 -0.088367156
N 0.763536349 0.201883927 -0.102084153
C 0.772407036 0.102289632 -0.082553018
N 0.881873334 0.096522213 -0.083883006
N 0.889377870 0.436514174 -0.070109758
C 0.897710368 0.670117404 -0.082553018
N 0.903477787 0.785351122 -0.083883006

- 4.14×10^{13} holes/cm^2

ATOMIC_POSITIONS crystal
N 0.000000000 0.000000000 -0.058584730
C 0.002446167 0.546932944 -0.068260685
N 0.000196244 0.663061832 -0.064634912
C -0.002110684 0.879861404 -0.059609403
C 0.544486777 0.997553833 -0.068260685
N 0.662865587 0.999803756 -0.064634912
C 0.881972087 1.002110684 -0.059609403
N 0.093234797 0.877007780 -0.033221356
C 0.100772580 0.769145715 -0.038430780
N 0.114662117 0.550052705 -0.060515442
N 0.122992220 0.216227018 -0.033221356
C 0.120138596 0.118027913 -0.059609403
N 0.214721256 0.115795756 -0.086721864
C 0.230854285 0.331626864 -0.038430780
N 0.241413551 0.441907669 -0.020320460
C 0.208866689 0.657484907 -0.038338920
N 0.200494118 0.758586449 -0.020320460
N 0.333333300 0.666666700 -0.034055464
C 0.342515093 0.551381883 -0.038338920

N 0.336938168 0.337134413 -0.064634912
C 0.329277837 0.225392784 -0.086547383
N 0.437558670 0.233284618 -0.105581492
C 0.453067056 0.455513223 -0.068260685
N 0.449947295 0.564609515 -0.060515442
C 0.448618117 0.791133311 -0.038338920
N 0.435390485 0.885337883 -0.060515442
N 0.558092331 0.799505882 -0.020320460
N 0.561765664 0.452698778 -0.078516587
C 0.549657506 0.340459383 -0.094377223
N 0.547301222 0.109066786 -0.078516587
C 0.659540617 0.209198022 -0.094377223
N 0.666666700 0.333333300 -0.098450600
C 0.668373136 0.899227420 -0.038430780
N 0.783772982 0.906765203 -0.033221356
N 0.795725950 0.562441330 -0.105581492
C 0.790801978 0.450342494 -0.094377223
N 0.766715382 0.204274050 -0.105581492
C 0.774607216 0.103885053 -0.086547383
N 0.884204244 0.098925501 -0.086721864
N 0.890933214 0.438234336 -0.078516587
C 0.896114947 0.670722163 -0.086547383
N 0.901074499 0.785278744 -0.086721864

- 6.21×10^{13} holes/cm^2
  ATOMIC_POSITIONS crystal
  N 0.000000000 0.000000000 -0.048726307
  C 0.004898328 0.559841267 -0.093516849
  N -0.002376351 0.666831386 -0.075176974
  C -0.007990091 0.876680499 -0.052548795
  C 0.554942940 0.995101672 -0.093516849
  N 0.669207738 1.002376351 -0.075176974
  C 0.884670591 1.007990091 -0.052548795
  N 0.091666273 0.868989995 -0.032885064
  C 0.102120637 0.767809570 -0.048743269
  N 0.118382119 0.563756146 -0.091749223
  N 0.131010005 0.222676280 -0.032885064
  C 0.123319501 0.115329409 -0.052548795
  N 0.217051832 0.103567604 -0.075736006
  C 0.232190430 0.334311065 -0.048743269
  N 0.240629581 0.449078156 -0.041018052
  C 0.212546413 0.662897551 -0.065683813
  N 0.208448576 0.759370419 -0.041018052

N 0.333333300 0.666666700 -0.063864461
C 0.337102449 0.549648964 -0.065683813
N 0.333168614 0.330792262 -0.075176974
C 0.329162499 0.212545529 -0.082263874
N 0.439640882 0.215209919 -0.094046770
C 0.440158733 0.445057060 -0.093516849
N 0.436243854 0.554626071 -0.091749223
C 0.450351036 0.787453587 -0.065683813
N 0.445373929 0.881617881 -0.091749223
N 0.550921844 0.791551424 -0.041018052
N 0.542464748 0.439079877 -0.111099482
C 0.543822090 0.328827150 -0.104782379
N 0.560920123 0.103384769 -0.111099482
C 0.671172850 0.214994841 -0.104782379
N 0.666666700 0.333333300 -0.108289196
C 0.665688935 0.897879363 -0.048743269
N 0.777323720 0.908333727 -0.032885064
N 0.775569037 0.560359118 -0.094046770
C 0.785005159 0.456177910 -0.104782379
N 0.784790081 0.224430963 -0.094046770
C 0.787454471 0.116616970 -0.082263874
N 0.896432396 0.113484229 -0.075736006
N 0.896615231 0.457535252 -0.111099482
C 0.883383030 0.670837501 -0.082263874
N 0.886515771 0.782948168 -0.075736006

- 8.29×10^{13} holes/cm^2

  ATOMIC_POSITIONS crystal
  N 0.000000000 0.000000000 -0.067239719
  C 0.005420366 0.556458973 -0.101012262
  N -0.000219051 0.667035450 -0.087754116
  C -0.005664294 0.878325748 -0.070269804
  C 0.551038608 0.994579634 -0.101012262
  N 0.667254502 1.000219051 -0.087754116
  C 0.883990043 1.005664294 -0.070269804
  N 0.093334367 0.872638775 -0.049159921
  C 0.103206343 0.769623686 -0.061871305
  N 0.117688122 0.559715259 -0.097727047
  N 0.127361225 0.220695591 -0.049159921
  C 0.121674252 0.116009957 -0.070269804
  N 0.216069732 0.106779281 -0.093863898
  C 0.230376314 0.333582656 -0.061871305

N 0.239659566 0.447228970 -0.051258898
C 0.212311162 0.662032040 -0.073581865
N 0.207569403 0.760340434 -0.051258898
N 0.333333300 0.666666700 -0.071145571
C 0.337967960 0.550279223 -0.073581865
N 0.332964550 0.332745498 -0.087754116
C 0.327997523 0.215660638 -0.098408030
N 0.437950958 0.219083405 -0.111244844
C 0.443541027 0.448961392 -0.101012262
N 0.440284741 0.557972964 -0.097727047
C 0.449720777 0.787688838 -0.073581865
N 0.442027036 0.882311878 -0.097727047
N 0.552771030 0.792430597 -0.051258898
N 0.548974102 0.444857532 -0.115044012
C 0.545978835 0.332347732 -0.115047589
N 0.555142468 0.104116469 -0.115044012
C 0.667652268 0.213631003 -0.115047589
N 0.666666700 0.333333300 -0.117703546
C 0.666417344 0.896793657 -0.061871305
N 0.779304409 0.906665633 -0.049159921
N 0.781132447 0.562049042 -0.111244844
C 0.786368997 0.454021165 -0.115047589
N 0.780916595 0.218867553 -0.111244844
C 0.784339362 0.112336886 -0.098408030
N 0.893220719 0.109290452 -0.093863898
N 0.895883531 0.451025898 -0.115044012
C 0.887663114 0.672002477 -0.098408030
N 0.890709548 0.783930268 -0.093863898

- 1.04×10^{14} holes/cm^2

  ATOMIC_POSITIONS (crystal)
  N 0.000000000 0.000000000 -0.077517570
  C 0.005371949 0.554762147 -0.105034215
  N 0.000651344 0.666868636 -0.094462596
  C -0.004371187 0.879100617 -0.079758615
  C 0.549390197 0.994628051 -0.105034215
  N 0.666217294 0.999348656 -0.094462596
  C 0.883471806 1.004371187 -0.079758615
  N 0.094810439 0.874797866 -0.058305868
  C 0.103916743 0.770650518 -0.069168599
  N 0.116770743 0.557494077 -0.100660071
  N 0.125202134 0.220012572 -0.058305868

C 0.120899383 0.116528194 -0.079758615
N 0.216677852 0.108599845 -0.102572144
C 0.229349482 0.333266224 -0.069168599
N 0.238955723 0.446103009 -0.056980930
C 0.212015222 0.661527052 -0.077750838
N 0.207147285 0.761044277 -0.056980930
N 0.333333300 0.666666700 -0.074885955
C 0.338472948 0.550488270 -0.077750838
N 0.333131364 0.333782706 -0.094462596
C 0.328253655 0.217325633 -0.106014839
N 0.438743937 0.221513076 -0.118317296
C 0.445237853 0.450609803 -0.105034215
N 0.442505923 0.559276769 -0.100660071
C 0.449511730 0.787984778 -0.077750838
N 0.440723231 0.883229257 -0.100660071
N 0.553896991 0.792852715 -0.056980930
N 0.551668597 0.446582659 -0.117396429
C 0.547469321 0.333928593 -0.119653650
N 0.553417341 0.105085837 -0.117396429
C 0.666071407 0.213540628 -0.119653650
N 0.666666700 0.333333300 -0.121914394
C 0.666733776 0.896083257 -0.069168599
N 0.779987428 0.905189561 -0.058305868
N 0.782769139 0.561256063 -0.118317296
C 0.786459372 0.452530679 -0.119653650
N 0.778486924 0.217230861 -0.118317296
C 0.782674367 0.110928023 -0.106014839
N 0.891400155 0.108078009 -0.102572144
N 0.894914163 0.448331403 -0.117396429
C 0.889071977 0.671746345 -0.106014839
N 0.891921991 0.783322148 -0.102572144

- 1.24×10^{14} holes/cm^2

  ATOMIC_POSITIONS (crystal)
  N 0.000000000 0.000000000 -0.085358209
  C 0.004952522 0.555346203 -0.112204089
  N 0.001236329 0.667956007 -0.102657376
  C -0.003634685 0.879632256 -0.087600922
  C 0.550393679 0.995047478 -0.112204089
  N 0.666719678 0.998763671 -0.102657376
  C 0.883266941 1.003634685 -0.087600922
  N 0.096922943 0.876204203 -0.067522217

C 0.105946859 0.772578717 -0.078782884
N 0.116017841 0.558197701 -0.109041878
N 0.123795797 0.220718739 -0.067522217
C 0.120367744 0.116733059 -0.087600922
N 0.217392222 0.108674577 -0.108634390
C 0.227421283 0.333368140 -0.078782884
N 0.236433990 0.446061743 -0.067668736
C 0.213022832 0.663115859 -0.087898367
N 0.209627752 0.763566010 -0.067668736
N 0.333333300 0.666666700 -0.085708208
C 0.336884141 0.549907072 -0.087898367
N 0.332043993 0.333280322 -0.102657376
C 0.328427507 0.216910902 -0.111920392
N 0.439922284 0.221257533 -0.121465028
C 0.444653797 0.449606321 -0.112204089
N 0.441802299 0.557820242 -0.109041878
C 0.450092928 0.786977168 -0.087898367
N 0.442179758 0.883982159 -0.109041878
N 0.553938257 0.790372248 -0.067668736
N 0.551733670 0.445232607 -0.121902960
C 0.547957093 0.333285763 -0.123161506
N 0.554767393 0.106500964 -0.121902960
C 0.666714237 0.214671232 -0.123161506
N 0.666666700 0.333333300 -0.124495343
C 0.666631860 0.894053141 -0.078782884
N 0.779281261 0.903077057 -0.067522217
N 0.781335249 0.560077716 -0.121465028
C 0.785328768 0.452042907 -0.123161506
N 0.778742467 0.218664751 -0.121465028
C 0.783089098 0.111516606 -0.111920392
N 0.891325423 0.108717647 -0.108634390
N 0.893499036 0.448266330 -0.121902960
C 0.888483394 0.671572493 -0.111920392
N 0.891282353 0.782607778 -0.108634390

- 1.45×10^{14} holes/cm^2

  ATOMIC_POSITIONS (crystal)
  N 0.000000000 0.000000000 -0.092630979
  C 0.004277078 0.555135460 -0.116898842
  N 0.001760832 0.668555769 -0.109153097
  C -0.002723828 0.880317789 -0.094654101
  C 0.550858381 0.995722922 -0.116898842

N 0.666794937 0.998239168 -0.109153097
C 0.883041618 1.002723828 -0.094654101
N 0.099354618 0.878076460 -0.075948856
C 0.107874655 0.774500147 -0.086997068
N 0.114937042 0.557901000 -0.114224921
N 0.121923540 0.221278157 -0.075948856
C 0.119682211 0.116958382 -0.094654101
N 0.218385107 0.109312885 -0.113466416
C 0.225499853 0.333374509 -0.086997068
N 0.233919960 0.445629242 -0.076425038
C 0.213758449 0.664115799 -0.095397324
N 0.211709282 0.766080040 -0.076425038
N 0.333333300 0.666666700 -0.093552551
C 0.335884201 0.549642750 -0.095397324
N 0.331444231 0.333205063 -0.109153097
C 0.328997704 0.217188956 -0.116508979
N 0.440882137 0.221599154 -0.123624963
C 0.444864540 0.449141619 -0.116898842
N 0.442099000 0.557036144 -0.114224921
C 0.450357250 0.786241551 -0.095397324
N 0.442963856 0.885062958 -0.114224921
N 0.554370758 0.788290718 -0.076425038
N 0.552307064 0.444674428 -0.123969124
C 0.548471430 0.333168191 -0.125276022
N 0.555325572 0.107632537 -0.123969124
C 0.666831809 0.215303139 -0.125276022
N 0.666666700 0.333333300 -0.126047497
C 0.666625491 0.892125345 -0.086997068
N 0.778721843 0.900645382 -0.075948856
N 0.780717017 0.559117863 -0.123624963
C 0.784696861 0.451528570 -0.125276022
N 0.778400846 0.219282983 -0.123624963
C 0.782811044 0.111808749 -0.116508979
N 0.890687115 0.109072223 -0.113466416
N 0.892367463 0.447692936 -0.123969124
C 0.888191251 0.671002296 -0.116508979
N 0.890927777 0.781614893 -0.113466416

- 1.57×10^{14} holes/cm^2

  ATOMIC_POSITIONS (crystal)
  N 0.000000000 0.000000000 -0.097598345
  C 0.003953170 0.554879324 -0.119401393

N 0.002158521 0.668835129 -0.113038882
C -0.002015316 0.880860554 -0.099351359
C 0.550926153 0.996046830 -0.119401393
N 0.666676609 0.997841479 -0.113038882
C 0.882875872 1.002015316 -0.099351359
N 0.100935911 0.879492600 -0.081315347
C 0.109069644 0.775733465 -0.091938997
N 0.114387242 0.557630203 -0.116784657
N 0.120507400 0.221443311 -0.081315347
C 0.119139446 0.117124128 -0.099351359
N 0.218948294 0.109885587 -0.116725682
C 0.224266535 0.333336178 -0.091938997
N 0.232392587 0.445238902 -0.081348130
C 0.214200681 0.664663552 -0.099405041
N 0.212846313 0.767607413 -0.081348130
N 0.333333300 0.666666700 -0.097663326
C 0.335336448 0.549537231 -0.099405041
N 0.331164871 0.333323391 -0.113038882
C 0.329380098 0.217569798 -0.119367242
N 0.441471379 0.222022567 -0.124763370
C 0.445120676 0.449073847 -0.119401393
N 0.442369797 0.556757142 -0.116784657
C 0.450462769 0.785799319 -0.099405041
N 0.443242858 0.885612758 -0.116784657
N 0.554761098 0.787153687 -0.081348130
N 0.552757732 0.444618528 -0.124803154
C 0.548865731 0.333323933 -0.126242857
N 0.555381472 0.108139104 -0.124803154
C 0.666676067 0.215541699 -0.126242857
N 0.666666700 0.333333300 -0.126583123
C 0.666663822 0.890930356 -0.091938997
N 0.778556689 0.899064089 -0.081315347
N 0.780551189 0.558528621 -0.124763370
C 0.784458301 0.451134269 -0.126242857
N 0.777977433 0.219448811 -0.124763370
C 0.782430202 0.111810301 -0.119367242
N 0.890114413 0.109062708 -0.116725682
N 0.891860896 0.447242268 -0.124803154
C 0.888189699 0.670619902 -0.119367242
N 0.890937292 0.781051706 -0.116725682

- 1.61×10^{14} holes/cm^2

ATOMIC_POSITIONS (crystal)
N 0.000000000 0.000000000 -0.098586557
C 0.003798205 0.554955964 -0.120185605
N 0.002135724 0.668880470 -0.113963775
C -0.001852456 0.880978213 -0.100354519
C 0.551157758 0.996201795 -0.120185605
N 0.666744748 0.997864276 -0.113963775
C 0.882830671 1.001852456 -0.100354519
N 0.101389876 0.879761626 -0.082709024
C 0.109391912 0.776018993 -0.093319283
N 0.114228780 0.557811403 -0.117792554
N 0.120238374 0.221628250 -0.082709024
C 0.119021787 0.117169329 -0.100354519
N 0.219110869 0.110059689 -0.117259385
C 0.223981007 0.333372919 -0.093319283
N 0.231921872 0.445204114 -0.083020035
C 0.214379062 0.664914651 -0.100864468
N 0.213282241 0.768078128 -0.083020035
N 0.333333300 0.666666700 -0.099227003
C 0.335085349 0.549464511 -0.100864468
N 0.331119530 0.333255252 -0.113963775
C 0.329515996 0.217637781 -0.119901618
N 0.441527036 0.222026139 -0.124902057
C 0.445044036 0.448842242 -0.120185605
N 0.442188597 0.556417478 -0.117792554
C 0.450535489 0.785620938 -0.100864468
N 0.443582522 0.885771220 -0.117792554
N 0.554795886 0.786717759 -0.083020035
N 0.552657889 0.444435628 -0.125183786
C 0.548848883 0.333257476 -0.126430430
N 0.555564372 0.108222161 -0.125183786
C 0.666742524 0.215591307 -0.126430430
N 0.666666700 0.333333300 -0.126612911
C 0.666627081 0.890608088 -0.093319283
N 0.778371750 0.898610124 -0.082709024
N 0.780499105 0.558472964 -0.124902057
C 0.784408693 0.451151117 -0.126430430
N 0.777973861 0.219500895 -0.124902057
C 0.782362219 0.111878217 -0.119901618
N 0.889940311 0.109051179 -0.117259385
N 0.891777839 0.447342111 -0.125183786
C 0.888121783 0.670484004 -0.119901618
N 0.890948821 0.780889131 -0.117259385

- 1.66×10^{14} holes/cm^2

  ATOMIC_POSITIONS crystal
  N 0.000000446 0.000000665 -0.104636001
  C 0.002403386 0.554629185 -0.120870056
  N 0.001747521 0.668430900 -0.116806045
  C -0.000937085 0.881499080 -0.105895360
  C 0.552223044 0.997594281 -0.120870003
  N 0.666682640 0.998253461 -0.116806071
  C 0.882436027 1.000936466 -0.105895310
  N 0.104380100 0.881987575 -0.091131244
  C 0.111083063 0.777751669 -0.100080812
  N 0.112483510 0.557745823 -0.118982618
  N 0.118011070 0.222390881 -0.091131208
  C 0.118501769 0.117563244 -0.105895482
  N 0.220823638 0.111876890 -0.119047088
  C 0.222250161 0.333335177 -0.100080764
  N 0.228854658 0.444244052 -0.091138327
  C 0.214842453 0.665780985 -0.105901221
  N 0.215390967 0.771145104 -0.091138193
  N 0.333332528 0.666666976 -0.104682872
  C 0.334216993 0.549061003 -0.105901059
  N 0.331569324 0.333316583 -0.116806114
  C 0.330905228 0.218839706 -0.120948047
  N 0.442020304 0.222829065 -0.123815250
  C 0.445370278 0.447776084 -0.120869970
  N 0.442256175 0.554735679 -0.118982998
  C 0.450939595 0.785153970 -0.105900784
  N 0.445265758 0.887523828 -0.118983323
  N 0.555755345 0.784610717 -0.091138407
  N 0.552516324 0.443802503 -0.123647849
  C 0.548779253 0.333317651 -0.124753800
  N 0.556196178 0.108710567 -0.123647859
  C 0.666682700 0.215463271 -0.124753836
  N 0.666666278 0.333333738 -0.124391305
  C 0.666666330 0.888913862 -0.100080497
  N 0.777607858 0.895620118 -0.091131293
  N 0.780808591 0.557981216 -0.123815381
  C 0.784540605 0.451218784 -0.124753865
  N 0.777171943 0.219190387 -0.123815223
  C 0.781163007 0.112066267 -0.120947998
  N 0.888120962 0.108948639 -0.119047421
  N 0.891283850 0.447483804 -0.123647727
  C 0.887935546 0.669097132 -0.120948078
  N 0.891051681 0.779173006 -0.119047375

- 2.07×10^{14} holes/cm^2

  ATOMIC_POSITIONS crystal
  N 0.000000446 0.000000665 -0.115669578
  C 0.000927334 0.553562597 -0.125931083
  N 0.001046901 0.667717322 -0.125066079
  C -0.000357329 0.882364390 -0.116641315
  C 0.552632508 0.999070333 -0.125931030
  N 0.666669683 0.998954081 -0.125066105
  C 0.882721582 1.000356710 -0.116641265
  N 0.108079468 0.884981404 -0.106890524
  C 0.113030613 0.779695456 -0.113867543
  N 0.110870085 0.556695434 -0.123857837
  N 0.115017241 0.223096420 -0.106890488
  C 0.117636459 0.117277689 -0.116641437
  N 0.222460536 0.112480503 -0.123862563
  C 0.220306374 0.333338940 -0.113867495
  N 0.225247731 0.443572218 -0.106891338
  C 0.215700113 0.666309006 -0.116641632
  N 0.218326060 0.774752031 -0.106891204
  N 0.333332528 0.666666976 -0.115675632
  C 0.333688972 0.549390642 -0.116641470
  N 0.332282902 0.333329540 -0.125066148
  C 0.332404186 0.219292727 -0.125931650
  N 0.442880179 0.222895102 -0.125605641
  C 0.446436866 0.447366620 -0.125930997
  N 0.443306564 0.554172642 -0.123858217
  C 0.450609956 0.784296310 -0.116641195
  N 0.445828795 0.889137253 -0.123858542
  N 0.556427179 0.781675624 -0.106891418
  N 0.553315731 0.443770532 -0.125606845
  C 0.549286279 0.333331332 -0.126580283
  N 0.556228149 0.109541943 -0.125606855
  C 0.666669019 0.215956616 -0.126580319
  N 0.666666278 0.333333738 -0.125584925
  C 0.666662567 0.886966312 -0.113867228
  N 0.776902319 0.891920750 -0.106890573
  N 0.780014752 0.557121341 -0.125605772
  C 0.784047260 0.450711758 -0.126580348
  N 0.777105906 0.219984226 -0.125605614
  C 0.780709986 0.113112205 -0.125931601
  N 0.887517349 0.109981923 -0.123862896

N 0.890452474 0.446684397 -0.125606723
C 0.886889608 0.667598174 -0.125931681
N 0.890018397 0.777536108 -0.123862850

- 2.40×10^{14} holes/cm^2

ATOMIC_POSITIONS crystal
N 0.000000000 0.000000000 -0.124288038
C -0.000032080 0.553184818 -0.127571674
N -0.000061562 0.666641410 -0.129999104
C -0.000012294 0.882599038 -0.125320757
C 0.553216900 1.000032080 -0.127571674
N 0.666702970 1.000061562 -0.129999104
C 0.882611333 1.000012294 -0.125320757
N 0.109641078 0.886083717 -0.124075606
C 0.113386205 0.780048677 -0.127197736
N 0.109772015 0.556928571 -0.124849812
N 0.113916283 0.223557361 -0.124075606
C 0.117400962 0.117388667 -0.125320757
N 0.223558539 0.113874022 -0.123912080
C 0.219951323 0.333337528 -0.127197736
N 0.223747490 0.443139637 -0.124200448
C 0.215923468 0.666603908 -0.125832428
N 0.219392146 0.776252510 -0.124200448
N 0.333333300 0.666666700 -0.124792567
C 0.333396092 0.549319661 -0.125832428
N 0.333358590 0.333297030 -0.129999104
C 0.333355369 0.219897940 -0.126935064
N 0.443124552 0.223691683 -0.123596334
C 0.446815182 0.446783100 -0.127571674
N 0.443071429 0.552843545 -0.124849812
C 0.450680339 0.784076532 -0.125832428
N 0.447156455 0.890227985 -0.124849812
N 0.556860363 0.780607854 -0.124200448
N 0.552845869 0.443037487 -0.124551252
C 0.549301065 0.333355814 -0.125279650
N 0.556962513 0.109808282 -0.124551252
C 0.666644186 0.215945152 -0.125279650
N 0.666666700 0.333333300 -0.124094489
C 0.666662472 0.886613795 -0.127197736
N 0.776442639 0.890358922 -0.124075606
N 0.780567131 0.556875448 -0.123596334
C 0.784054848 0.450698935 -0.125279650

N 0.776308317 0.219432869 -0.123596334
C 0.780102060 0.113457427 -0.126935064
N 0.886125978 0.109684516 -0.123912080
N 0.890191718 0.447154131 -0.124551252
C 0.886542573 0.666644631 -0.126935064
N 0.890315484 0.776441461 -0.123912080

- 2.48×10^{14} holes/cm^2

ATOMIC_POSITIONS crystal
N 0.000000000 0.000000000 -0.124998564
C -0.000020482 0.553178773 -0.128154259
N 0.000011097 0.666684980 -0.130830872
C 0.000066167 0.882731640 -0.126163255
C 0.553199255 1.000020482 -0.128154259
N 0.666673882 0.999988903 -0.130830872
C 0.882665473 0.999933833 -0.126163255
N 0.109719463 0.886180896 -0.124981004
C 0.113488742 0.780150193 -0.128084888
N 0.109806752 0.556989012 -0.125119080
N 0.113819104 0.223538567 -0.124981004
C 0.117268360 0.117334527 -0.126163255
N 0.223460613 0.113892750 -0.124764877
C 0.219849807 0.333338548 -0.128084888
N 0.223705848 0.443172245 -0.124988924
C 0.215994579 0.666628550 -0.126363729
N 0.219466396 0.776294152 -0.124988924
N 0.333333300 0.666666700 -0.125246368
C 0.333371450 0.549366130 -0.126363729
N 0.333315020 0.333326118 -0.130830872
C 0.333288350 0.219868492 -0.127949016
N 0.443107525 0.223665882 -0.124828246
C 0.446821227 0.446800745 -0.128154259
N 0.443010988 0.552817841 -0.125119080
C 0.450633870 0.784005421 -0.126363729
N 0.447182159 0.890193248 -0.125119080
N 0.556827755 0.780533604 -0.124988924
N 0.552820622 0.442954992 -0.125080836
C 0.549323056 0.333292604 -0.126263018
N 0.557045008 0.109865529 -0.125080836
C 0.666707396 0.216030352 -0.126263018
N 0.666666700 0.333333300 -0.125127813
C 0.666661452 0.886511258 -0.128084888

N 0.776461433 0.890280537 -0.124981004
N 0.780558357 0.556892475 -0.124828246
C 0.783969648 0.450676944 -0.126263018
N 0.776334118 0.219441643 -0.124828246
C 0.780131508 0.113419856 -0.127949016
N 0.886107250 0.109567862 -0.124764877
N 0.890134471 0.447179378 -0.125080836
C 0.886580144 0.666711650 -0.127949016
N 0.890432138 0.776539387 -0.124764877

References

(1) Hohenberg, P.; Kohn, W. Inhomogeneous electron gas. *Physical review* **1964**, *136*, B864.

(2) Kohn, W.; Sham, L. J. Self-Consistent Equations Including Exchange and Correlation Effects. *Phys. Rev.* **1965**, *140*, A1133–A1138.

(3) Giannozzi, P.; Baroni, S.; Bonini, N.; Calandra, M.; Car, R.; Cavazzoni, C.; Ceresoli, D.; Chiarotti, G. L.; Cococcioni, M.; Dabo, I.; others QUANTUM ESPRESSO: a modular and open-source software project for quantum simulations of materials. *Journal of physics: Condensed matter* **2009**, *21*, 395502.

(4) Giannozzi, P.; Andreussi, O.; Brumme, T.; Bunau, O.; Nardelli, M. B.; Calandra, M.; Car, R.; Cavazzoni, C.; Ceresoli, D.; Cococcioni, M.; others Advanced capabilities for materials modelling with Quantum ESPRESSO. *Journal of physics: Condensed matter* **2017**, *29*, 465901.

(5) Perdew, J. P.; Burke, K.; Ernzerhof, M. Generalized gradient approximation made simple. *Physical review letters* **1996**, *77*, 3865.

(6) Prandini, G.; Marrazzo, A.; Castelli, I. E.; Mounet, N.; Marzari, N. Precision and efficiency in solid-state pseudopotential calculations. *npj Computational Materials* **2018**, *4*, 72.

(7) Vanderbilt, D. Soft self-consistent pseudopotentials in a generalized eigenvalue formalism. *Physical review B* **1990**, *41*, 7892.

(8) Brumme, T.; Calandra, M.; Mauri, F. Electrochemical doping of few-layer ZrNCl from first principles: Electronic and structural properties in field-effect configuration. *Physical Review B* **2014**, *89*, 245406.

(9) Sohier, T.; Calandra, M.; Mauri, F. Density functional perturbation theory for gated two-dimensional heterostructures: Theoretical developments and application to flexural phonons in graphene. *Physical Review B* **2017**, *96*, 075448.

(10) Re Fiorentin, M.; Risplendi, F.; Palummo, M.; Cicero, G. First-principles calculations of exciton radiative lifetimes in monolayer graphitic carbon nitride nanosheets: implications for photocatalysis. *ACS Applied Nano Materials* **2021**, *4*, 1985–1993.

(11) Zhou, H.; Xie, T.; Taniguchi, T.; Watanabe, K.; Young, A. F. Superconductivity in rhombohedral trilayer graphene. *Nature* **2021**, *598*, 434–438.

(12) Holleis, L.; Patterson, C. L.; Zhang, Y.; Vituri, Y.; Yoo, H. M.; Zhou, H.; Taniguchi, T.; Watanabe, K.; Berg, E.; Nadj-Perge, S.; others Nematicity and orbital depairing in superconducting Bernal bilayer graphene. *Nature Physics* **2025**, 1–7.
