# Supplementary material for: Interplay of Superconductivity, Ferromagnetism, and Half-Metallicity in Gated Single-Layer g‑C3N4
Source: J Phys Chem Lett. 2025 Jun 3;16(23):5739–44. doi: 10.1021/acs.jpclett.5c01013 (PMC12169656; doi:10.1021/acs.jpclett.5c01013)
Supplement: Supplementary file 2 [file jz5c01013_si_002.pdf]

jz-2025-010136.R1

Name: Peer Review Information for "Interplay of Superconductivity, Ferromagnetism and Half-Metallicity in Gated Single-Layer g-C₃N₄."

First Round of Reviewer Comments

Reviewer: 1

Comments to the Author

The present manuscript by Pietro Nicolò Brangi et al. investigates the electronic and magnetic properties of field-effect hole-doped graphitic carbon nitride (g-C_3N_4) using first-principles calculations. The authors show that increasing the doping level progressively depletes the long-range electronic pairs forming flat bands, resulting in a rich phase diagram encompassing magnetic and superconducting phases. The study is both systematic and interesting. In particular, it introduces the intriguing possibility of realizing flat-band-driven correlated phases in a material with high density (atomic scale) — potentially enabling magnetic and superconducting states at significantly elevated temperature scales. I am inclined to recommend the manuscript for publication in The Journal of Physical Chemistry Letters. However, before making a recommendation, there are a few questions that I would like the authors to address.

1. The corrugation of the structure under hole doping appears to play a key role in the calculation. In a field-effect transistor setup, gating not only modulates the carrier density but also introduces an out-of-plane displacement field—known to have profound effects in Bernal-stacked bilayer and rhombohedral multilayer graphene systems. Could the authors discuss whether such a displacement field might significantly influence the band structure or phase diagram in g-C_3N_4?

2. The manuscript evaluates magnetism based on spin polarization. However, in graphene, orbital magnetization arising from Berry curvature effects can dominate and even exceed the spin contribution. Could the authors estimate or comment on the orbital magnetization in g-C_3N_4 and its potential role?

3. The authors report an insulating phase at integer filling (1 hole per 42-atom cell). Given that a single hole does not fully occupy a band, is this a Mott-like insulator? If so, why is it identified as a ferromagnetic insulator? In such cases, one might expect antiferromagnetic order instead. Could the authors clarify the nature and mechanism of magnetism in the insulating phase?

4. The term "half-metallic state" is used in the manuscript. For clarity, could the authors explicitly define this term in the context of their calculations?

5. The manuscript identifies superconducting states, yet several aspects remain unclear. What is the pairing symmetry of the superconducting order parameter? The emergence of superconductivity from a ferromagnetic metal is highly unconventional. Since such systems generally lack favorable nesting conditions for Cooper pairing, have the authors incorporated this into their analysis? In graphene systems, valley degrees of freedom (isospins) significantly influence the superconducting properties. Is there a similar valley-like structure in the band topology of g-C_3N_4?

6. At the reported high carrier densities, the relevant energy scales in g-C_3N_4 are roughly two orders of magnitude larger than in twisted bilayer or rhombohedral graphene. One might therefore expect a substantial enhancement in the superconducting transition temperature, possibly on the order of 100–200 K. Yet the reported Tc is comparable to existing graphene-based superconductors. Could the authors provide a physical explanation for this discrepancy? Another note that the latest results from rhombohedral six-layer graphene (arXiv:2504.05129) already show a superconducting transition at ~1 K.

Author's Response to Peer Review Comments:

Reply to the Referee’s comments

May 12, 2025

Dear Editor, find enclosed the reply to the Referee’s questions. As we carefully addressed all the points raised by the Referee, we believe that the paper is now ready to be published in J. Phys. Chem.
Lett.

Regards,

Pietro Brangi on behalf of all the authors.

We thank the referee for appreciating our work and considering it for publication in The Journal of Physical Chemistry Letters. We also thank him for raising some interesting points that we address here systematically:

1. *The corrugation of the structure under hole doping appears to play a key role in the calculation. In a field-effect transistor setup, gating not only modulates the carrier density but also introduces an out-of-plane displacement field—known to have profound effects in Bernal-stacked bilayer and rhombohedral multilayer graphene systems. Could the authors discuss whether such a displacement field might significantly influence the band structure or phase diagram in g-C_3N_4?*

   Our calculations include both the effect of charging and the effect of the displacement field as we use the Field Effect Transistor (FET) setup, as developed by some of us in Ref. [1]. In the experiment, as in our calculation, both effects are present at the same time. In order to underline the effect of the displacement field induced symmetry breaking and the consequent inhomogeneous charging of the layer, we compare our calculation with a simpler one performed with a uniform doping and no external electric field at n_h = 1 hole/ 42 atoms u.c.. The latter calculation neglects the effects of the displacement field both on the charge dishomogeneity in the layer and in the breaking of inversion symmetry along z. The results are included in a new section in the SI (Sec. S6).

   The results demonstrate that the displacement field is the dominant effect, as suggested by the Referee and as expected from our calculation.

   The band structure and magnetization density of the uniformly doped case (at the doping level of n_h = 1 hole/ 42 atoms u.c.) are reported in SI Sec. S6. More specifically, the displacement field splits the two top valence bands that are degenerate at Γ in the undoped case, which in turn determines structural rearrangements and the separation in energy of the first spin polarized band with respect to the rest of the lone-pair-like manifold. At the doping level of n_h = 1 hole/ 42 atoms u.c., at which we investigate the effect of the displacement field, this also results in having a metallic ferromagnetic phase as opposed to the insulating ferromagnetic one found in the FET configuration. The wavefunction associated to the highest occupied band also changes quite remarkably from being delocalized on several lone pairs (in the uniform doping case) to being localized on the lone pairs sitting closer to the gate. This is due to the electrostatic effect of the gate which attracts the lone pairs carrying the positive charge.

2. *The manuscript evaluates magnetism based on spin polarization. However, in graphene, orbital magnetization arising from Berry curvature effects can dominate and even exceed*

the spin contribution. Could the authors estimate or comment on the orbital magnetization in g-C_3N_4 and its potential role?

We computed the orbital magnetization of the cell with the method developed in Ref. [2] at the doping level of $n_h = 8.29 \times 10^{13}$ holes/cm^2 and we found it to be negligible (of the order of $10^{-3}\mu_B$, three orders of magnitude less than the spin magnetization). We added a paragraph to include this result in the main paper:

Finally, We computed the orbital magnetization with the method developed in Ref. [2] and we found it to be of the order of $10^{-3}\mu_B$ at $n_h = 8.29 \times 10^{13}$ holes/cm^2 and, thus, negligible.

3. *The authors report an insulating phase at integer filling (1 hole per 42-atom cell). Given that a single hole does not fully occupy a band, is this a Mott-like insulator? If so, why is it identified as a ferromagnetic insulator? In such cases, one might expect antiferromagnetic order instead. Could the authors clarify the nature and mechanism of magnetism in the insulating phase?*

   The magnetic state is spin polarized and the material displays a total cell magnetization of $1\mu_B$ at the doping level addressed by the referee. This means that spin up and spin down bands are non-degenerate (see Fig. 1 of the main article) and one hole fully occupies one spin polarized band. Thus, an insulating state can occur even for an odd number of electrons. At this charging, the physics is then very different from the one of a Mott like insulator, as it is the physics of a ferromagnetic band insulator.

   For completeness, we report that we also checked for other possible magnetic orderings such as antiferromagnetic ones, including Hubbard U of different values, but they turned out to be unstable and the ferromagnetic solution is by far the lowest energy one.

4. *The term "half-metallic state" is used in the manuscript. For clarity, could the authors explicitly define this term in the context of their calculations?*

   We thank the referee for this comment aiming at improving the readability of the paper. The standard definition of an half-metallic state is a metallic state in which the carriers belong to only one spin channel while the other spin channel remains insulating or poorly conducts. We added the following definition in the paper:

   (we label as half metal a system in which the carriers belongs only, or to a great majority, to one spin channel)

5. *The manuscript identifies superconducting states, yet several aspects remain unclear. What is the pairing symmetry of the superconducting order parameter? The emergence of superconductivity from a ferromagnetic metal is highly unconventional. Since such systems generally lack favorable nesting conditions for Cooper pairing, have the authors*

incorporated this into their analysis? In graphene systems, valley degrees of freedom (isospins) significantly influence the superconducting properties. Is there a similar valley-like structure in the band topology of g-C_3N_4?

The Referee is right in saying that the ferromagnetic pairing should be unconventional, as it should occur in a triplet state. In our paper, we suggested two options. Either the pairing is conventional and below T_c the system becomes nonmagnetic or it coexists with the magnetic state and it is nonconventional. However, there is no way in our theoretical framework to understand which one of the two instabilities (non magnetic singlet superconductor and magnetic spin triplet superconductor) will be the most stable one. Thus, we have left open the possibility for the two pairings to occur. What is really important is that the electron-phonon scattering remains strong even in the ferromagnetic state, which is anomalous.

The Referee is worried about the suppression of nesting in the ferromagnetic state. We underline that our analysis includes the nesting conditions for superconductivity and also its potential suppression in the magnetic state.

The Referee points out the possible role of the valley degrees of freedom. However, at odds with graphene, we do not have any valley-like structure in this system.

6. *At the reported high carrier densities, the relevant energy scales in g-C_3N_4 are roughly two orders of magnitude larger than in twisted bilayer or rhombohedral graphene. One might therefore expect a substantial enhancement in the superconducting transition temperature, possibly on the order of 100–200 K. Yet the reported Tc is comparable to existing graphene-based superconductors. Could the authors provide a physical explanation for this discrepancy? Another note that the latest results from rhombohedral six-layer graphene (arXiv:2504.05129) already show a superconducting transition at 1 K.*

The superconducting instability in rhombohedral graphene is intimately connected with the valley structure and the presence of intervalley scattering. This phenomenon is lacking in our case. The normal state in multilayer rhombohedral graphene is most likely a layer antiferromagnetic state [3], in our case it is a half-metallic ferromagnet. Thus, the situation is quite different.

In the case of a twisted bilayer graphene, the magnetic state is yet unknown, unfortunately. To authors knowledge, there is no evidence that it is ferromagnetic or half-metallic. Thus, even this case could be quite different and it is difficult to directly compare these results with ours.

Finally, we included in the manuscript the citation suggested by the Referee and we amended the paper correspondingly.

References

[1] Thibault Sohier, Matteo Calandra, and Francesco Mauri. Density functional perturbation theory for gated two-dimensional heterostructures: Theoretical developments and application to flexural phonons in graphene. *Physical Review B*, 96(7):075448, 2017.

[2] MG Lopez, David Vanderbilt, T Thonhauser, and Ivo Souza. Wannier-based calculation of the orbital magnetization in crystals. *Physical Review B—Condensed Matter and Materials Physics*, 85(1):014435, 2012.

[3] Y. Lee, D. Tran, K. Myhro, J. Velasco, N. Gillgren, C. N. Lau, Y. Barlas, J. M. Poumirol, D. Smirnov, and F. Guinea. Competition between spontaneous symmetry breaking and singleparticle gaps in trilayer graphene. *Nature Communications*, 5(1):5656, Dec 2014.
